# Genome-Wide Prediction and Analysis of 3D-Domain Swapped Proteins in the Human Genome from Sequence Information

**DOI:** 10.1371/journal.pone.0159627

**Published:** 2016-07-28

**Authors:** Atul Kumar Upadhyay, Ramanathan Sowdhamini

**Affiliations:** National Centre for Biological Sciences (TIFR), GKVK Campus, Bellary Road, Bangalore 560 065, India; International Centre for Genetic Engineering and Biotechnology (ICGEB), INDIA

## Abstract

3D-domain swapping is one of the mechanisms of protein oligomerization and the proteins exhibiting this phenomenon have many biological functions. These proteins, which undergo domain swapping, have acquired much attention owing to their involvement in human diseases, such as conformational diseases, amyloidosis, serpinopathies, proteionopathies etc. Early realisation of proteins in the whole human genome that retain tendency to domain swap will enable many aspects of disease control management. Predictive models were developed by using machine learning approaches with an average accuracy of 78% (85.6% of sensitivity, 87.5% of specificity and an MCC value of 0.72) to predict putative domain swapping in protein sequences. These models were applied to many complete genomes with special emphasis on the human genome. Nearly 44% of the protein sequences in the human genome were predicted positive for domain swapping. Enrichment analysis was performed on the positively predicted sequences from human genome for their domain distribution, disease association and functional importance based on Gene Ontology (GO). Enrichment analysis was also performed to infer a better understanding of the functional importance of these sequences. Finally, we developed hinge region prediction, in the given putative domain swapped sequence, by using important physicochemical properties of amino acids.

## Introduction

Computational methods for classification, annotation and prediction of biologically important questions are rapidly improving our knowledge of protein sequence-structure-function relationships. Detailed analysis of such relationships of protein improves our understanding of sequence features and its role in different biological pathways and diseases. Understanding the mechanism of protein oligomerization can influence many aspects of protein research such as drug discovery. This will also add to the existing pool of knowledge regarding amyloid formation and aggregation-related diseases.

**Fig 1:**
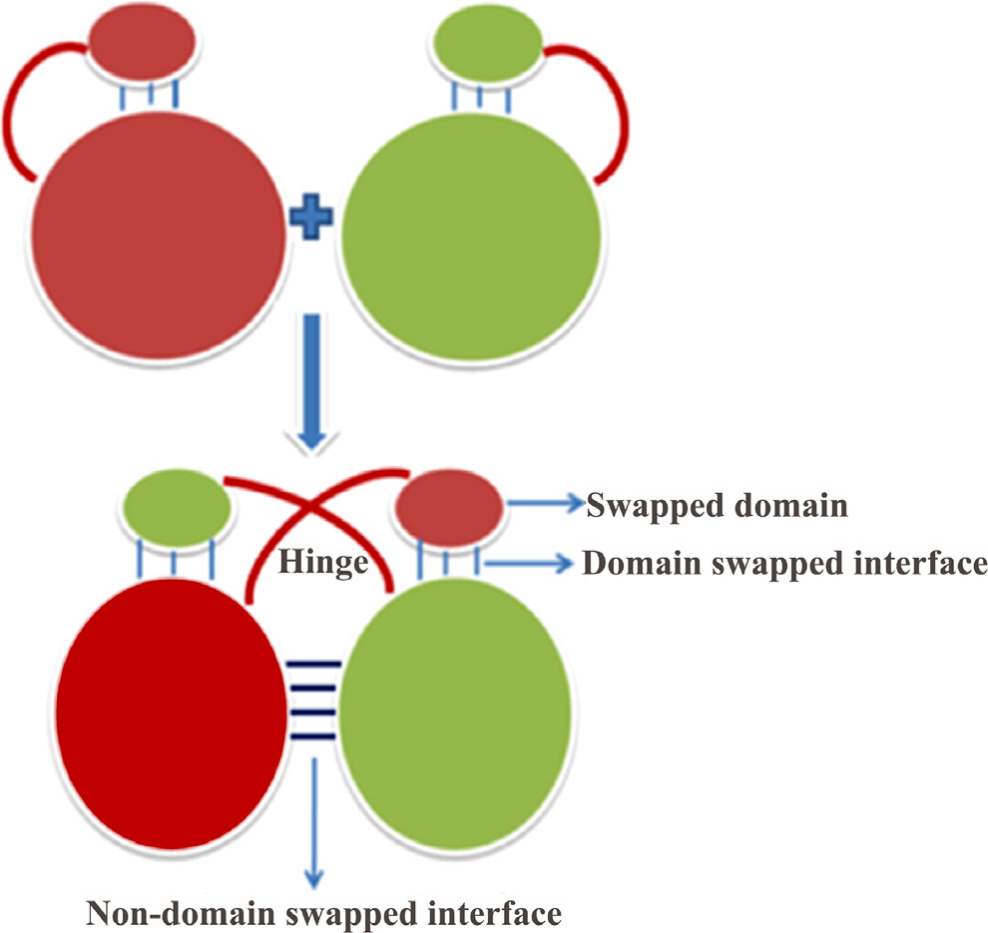
Cartoon representation of 3D-domain swapping. The region which connects the swapped domain with non-swapped domain part of the protein is known as “hinge region” and is marked in dark red color. There is a newly formed interface between non-domain swapped regions of the two monomeric units. The domain swapped interface is present both in monomer and the domain swapped molecule.

3D-domain swapping is one of the mechanisms of protein oligomerization and is also known to be involved in protein aggregation processes. 3D-domain swapping was first reported as a mechanism of oligomerisation in RNase A by Crestfield and coworkers n 1962 after lyophilization in 50% acetic acid [1]. The term 3D-domain swapping was first introduced in 1994, to explain a dimeric structure of diphtheria toxin protein [2]. In 3D-domain swapping, intertwined dimeric or oligomeric structures are formed by exchanging a structural element or complete domain of one of the monomeric units of the protein with the other. This exchange of structural element or complete domain results in gain of intermolecular interactions at the cost of similar intramolecular interactions. Peculiar characteristics of 3D-domain swapped oligomers which differentiate it from other side-by-side oligomers are ‘swapped domain’ and ‘hinge region’, please see Fig 1 for detail (Fig 1). Swapped domain is a structural part of the protein that gets exchanged between monomeric subunits, and this could be a whole globular domain or a supersecondary structure. Non-swapped domain is the counterpart of the swapped domain. The interface between swapped domain of one subunit and non-swapped domain of another subunit is known as “swapped domain interface (SDI)”. The newly formed interface between two non-swapped domains is referred as “non-swapped domain interface (NSI)”. The region of the protein, which connects swapped domain with non-swapped domain, is referred as the “hinge region”. Hinge region, being the most flexible region in the protein structure, plays an important role in the mechanism of domain swapping as it allows the movement of swapping domains. The hinge region mostly adopts loop conformation, but rarely also found as secondary structures based on the composition of the amino acids present in the hinge region sequences. There are several examples to show the importance of hinge region in domain swapping: for instance, shortening of hinge region increases the propensity of domain swapping by making it harder for the protein chain to fold back on itself [3]. Other condition for increasing the probability of domain swapping is by introducing more flexibility into hinge region either by mutation or by lengthening it. For example, in case of Chymotrypsin inhibitor 2, insertion of polyglutamine in the hinge region resulted in domain swapped dimer and higher order oligomer formation [4].

Three classes of domain swapping viz., bonafide domain swapping (BDS), quasi-domain swapping (QDS) and candidates for 3D-domain swapping (CDS) have been identified by Eisenberg and others in 2002 [5]. Most important class is the bonafide domain swapping, where a protein molecule exists in monomeric as well as in domain-swapped form e.g. Cyanovirin-N is present as monomer [6] and also as a dimer [7]. Second class is the quasi-domain swapping where a protein is present in the oligomeric form, but only its close monomeric homologue (not its monomer) is present, e.g. human cystatin C dimer [8] and chicken cystatin monomer [9]. The third class includes the candidates for 3D-domain swapping, in which protein structure of domain swapped form is present, but their monomers or monomeric homologous are not present, e.g. phosphoenol pyruvate mutase dimer [10].

3D-domain swapping is further classified on the basis of position of the swapping domain in the protein structure into three groups viz., N-terminal domain swapping, C-terminal domain swapping and the less common central domain swapping.

Collection of such protein structures which are involved in 3D-domain swapping in the form of database is of great help to researchers for further analysis and detailed study of this phenomenon [11]. To date, approximately >2000 crystal structures of 3D-domain-swapped oligomers have been reported [12]. From the available literature, it is known that 3D-domain swapped molecules have great biological significance in deposition diseases or conformational diseases [13][14], involved in misfolding related diseases such as amyloidosis [15] [1], serinopathies [16] and proteinopathies [17]. 3D-domain swapping is also proved to be a mechanism for regulating biological functions and as an evolutionary strategy to create protein complexes [18].

In recent times, various computational and experimental studies were employed to understand the mechanism of 3D-domain swapping in detail. Sequence and structural features were used to predict 3D-domain swapping from protein sequence earlier [19]. 3D-domain swapping events were predicted in protein sequences by using a SVM-based classifier derived from sequence and structural features and an accuracy of 76.33 form training and 73.81 from test data [20] were obtained. As we discussed the importance of the hinge region in domain swapping, an attempt was made earlier by our group to identify the hinge region by using domain-swapped oligomers and their homologous structures given their three-dimensional coordinates [21]. A meta-analysis of a literature-curated dataset of human gene products, with structural information and involved in 3D-domain swapping, was also performed earlier to obtain insight about the functional repertoire, pathway associations and disease implications of proteins involved in 3D-domain swapping [22].

Here, we present a computational approach towards prediction and genome-wide analysis of 3D-domain swap protein from mere sequence information by using machine learning approaches such as Random Forest (RF) [23] and support vector machine (SVM) classifier. RF is used for the prediction, as a binary classifier, because of its ability to combine several random decision trees and achieving a high rate of accuracy, and also since it can be effectively applied to larger dataset. Further, RF and SVM models were used to classify the protein sequences at the whole genome level of human and few other genomes into 3D-domain swapping and non 3D-domain swapping sequences. Positively predicted sequences from human genome were analyzed in detail for their distribution at protein domain family level, biological pathways and the involvement of these proteins in diseases.

Due to diverse structural, functional, structural and pharmaceutical implications, the prediction of proteins with a potential to engage in 3D-domain swapping from mere sequence information would be a great advantage in identifying plausible drug targets for therapeutic control of such diseases. Technical limitations of structural elucidation of proteins at such high number, and in higher oligomeric conformations by using crystallography or NMR experiments set the need to develop computational methods. In this work, we have restricted our feature extraction only to sequence space and it is the first attempt of this kind. Finally, we predict hinge regions for the domain-swapped proteins by using a sliding window approach based on the peculiar physicochemical features of residues in the hinge region.

## Results

3D-domain swapped protein molecules are often associated with aggregation diseases or proteinopathies in humans. Till now, no comprehensive study has been reported to analyze proteins involved in 3D-domain swapping from a genome-wide perspective. We have performed the initial investigation of putative 3D-domain swapped proteins at the level of protein domains, Gene Ontology, KEGG pathways and Disease Ontology.

### Classification results by RF and SVM models

Training was performed on a dataset containing 2000 protein sequences comprising of positive (1000) and negative (1000) datasets. We have performed five-fold cross validation on testing dataset. The RF prediction model achieved an accuracy of 81.7% with 81.5% of sensitivity, 81.8% of specificity and a MCC value of 0.64. The SVM prediction model achieved an accuracy of 73.9% with 61.9% of sensitivity, 85.9% of specificity and MCC value of 0.61 (Table 1). We have also analyzed the problematic cases such as false positives and false negative ones. The reasons for these cases of false positives and false negatives could be like: (1) central loop swapped entries with missing residues, (2) small hinge region, (3) domain swapped oligomer with elaborate interface or (4) intertwined domain swapped proteins and multiple hinge regions. In case of central loop swapping with missing residues, multiple hinge regions are involved and the swapped loop region exhibits majority of the characteristics of the hinge region, posing severe challenge on the prediction. In another problematic case, where intertwined structure is present, multiple loops are present hence, it is hard to predict correctly.

**Table 1:** Prediction assessment of RF and SVM models on testing dataset.

S. No.	Parameters	RF	SVM
1	Accuracy	81.7%	73.9%
2	Sensitivity	81.5%	61.9%
3	Specificity	81.8%	85.9%
4	MCC value	0.64	0.61

### Prediction results on well-known dataset of 3DSwap and human aggregation-related proteins by RF and SVM models

Performance of prediction using an independent validation dataset of the well-known cases of 3D-domain swapping from 3DSwap database resulted in 100% accuracy (S1 Table). For another dataset of 136 aggregation-related, reviewed human protein sequences from UNIPROT, 99 were predicted as positive for 3D-domain swapping. Out of 99 positively predicted sequences, 50 have homologues in Protein Data Bank, with identity more than 90%, and 29 sequences have homologues of known structure with identity range of 30–90%. For the rest of the 20 sequences, there are no homologues in the Protein Structural Databank. Sequences which are predicted as domain swapping and have structural homologues were manually analyzed to confirm 3D-domain swapping in these structures.

**Fig 2:**
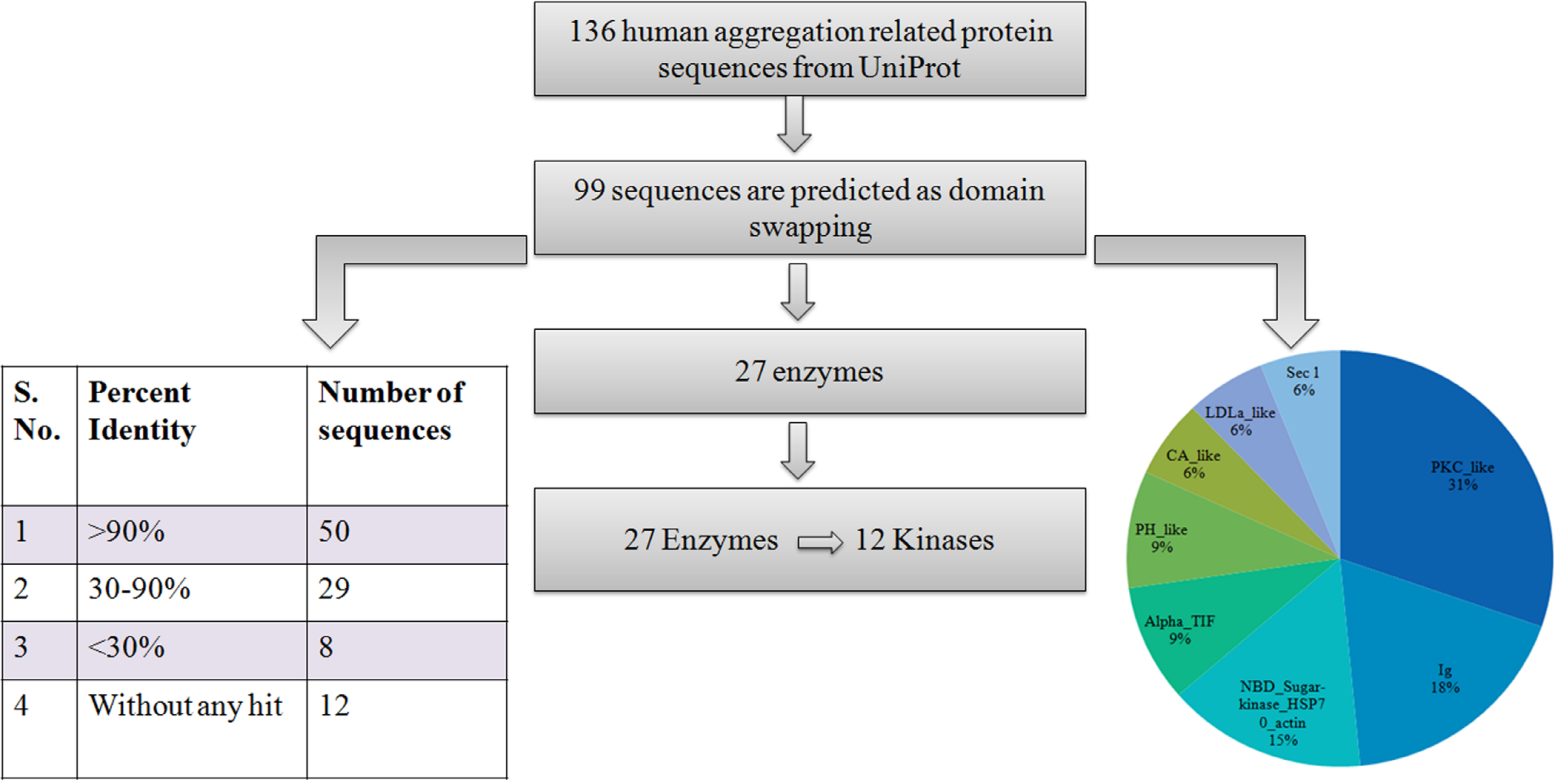
Workflow of 3D-domain swap prediction and analysis of aggregation-related sequences from the human genome. Out of 136 aggregation-related sequences, 99 were predicted as to be involved in domain swapping and its distribution in different Pfam domain families was plotted in the pie chart. All the positively predicted sequences were searched for their structural homologues.

Further analysis of these sequences reveals that 27 out of 99 sequences (3D-domain swapped predicted sequences) are enzymes and 12 of them contain kinase domains. We also analyzed the domain distribution of these positively predicted sequences (Fig 2). Pkc like superfamily, also known as PKC, is most populated in this dataset. The members of PKC Pfam family phosphorylate the hydroxyl groups of serine and threonine of other proteins and hence control the function of these proteins. Ig superfamily is the second most populated Pfam protein family, followed by NBD sugar kinase, amongst the positively predicted protein sequences.

### Prediction results on few of the non-model genomes from animal and plant kingdoms

RF and SVM models were applied on five plant genomes and three non-plant genomes including the human genome, for prediction of 3D-domain swapping. Common prediction results from both the models range from a minimum of 15.5% in *O*. *tenuiflorum* to maximum of 44.2% for *Homo sapiens* (considering only reviewed genome sequences) (Table 2). Consensus positive predictions on few of the plant genomes used in this study are *A*. *thaliana* (33.7%), *M*. *truncatula* (20.9%), *S*. *tumerosum* (36.5%), *S*. *lycopersicum* (25.5%), and *O*. *tenuiflorum* (15.5%). Non-plant genomes on which these prediction models were applied are *M*. *tuberculosis* (28.2%), *B*. *thuriensis* (43.3%) and *H*. *sapiens* (44.2%). The numbers in brackets represent percentage of consensus positive domain swapping sequences.

**Table 2:** 3D-domain swapping prediction results on different genomes.

S.No.	Genomes	Total reviewed sequences	Positive prediction by RF	Positive prediction by SVM	Consensus Positive prediction (RF and SVM)
1	*A*. *thaliana*	12033	7694 (64%)	6330 (53%)	4058 (33.7%)
2	*M*. *truncatula*	186	48 (26%)	66 (35%)	39 (20.9%)
3	*S*. *tumerosum*	400	208 (52%)	165 (41.3%)	146 (36.5%)
4	*S*. *lycopersicum*	423	183 (43%)	132 (31.2%)	108 (25.5%)
5	*O*. *tenuiflorum*	36841	9419 (26%)	7540 (21%)	5706 (15.5%)
6	*M*. *tuberculosis*	421	229 (54.3%)	134 (31.8%)	119 (28.2%)
7	*B*. *thuriensis*	393	246 (62.5%)	199 (50.6%)	170 (43.3%)
8	*H*. *sapiens*	20247	11507 (56.7%)	12396 (61.2%)	8945 (44.2%)

### Prediction results on the human proteome

We have performed 3D-domain swapping prediction on gene products in the human genome for reviewed and unreviewed entries, separately. Out of 20,247 reviewed entries in the human proteome, 57% of these (i.e., 11,507) are predicted as 3D-domain swapped by RF model and 61% (i.e., 12396) sequences are predicted as positive by SVM method, thereby identifying 44% of the sequences which were predicted positive by both the methods. In case of the entire set (reviewed and unreviewed) consisting of 115,830 sequences, 24% of these are predicted positive for domain swapping.

### Detailed analysis of 3D-domain swap predicted sequences of the human proteome

**Fig 3:**
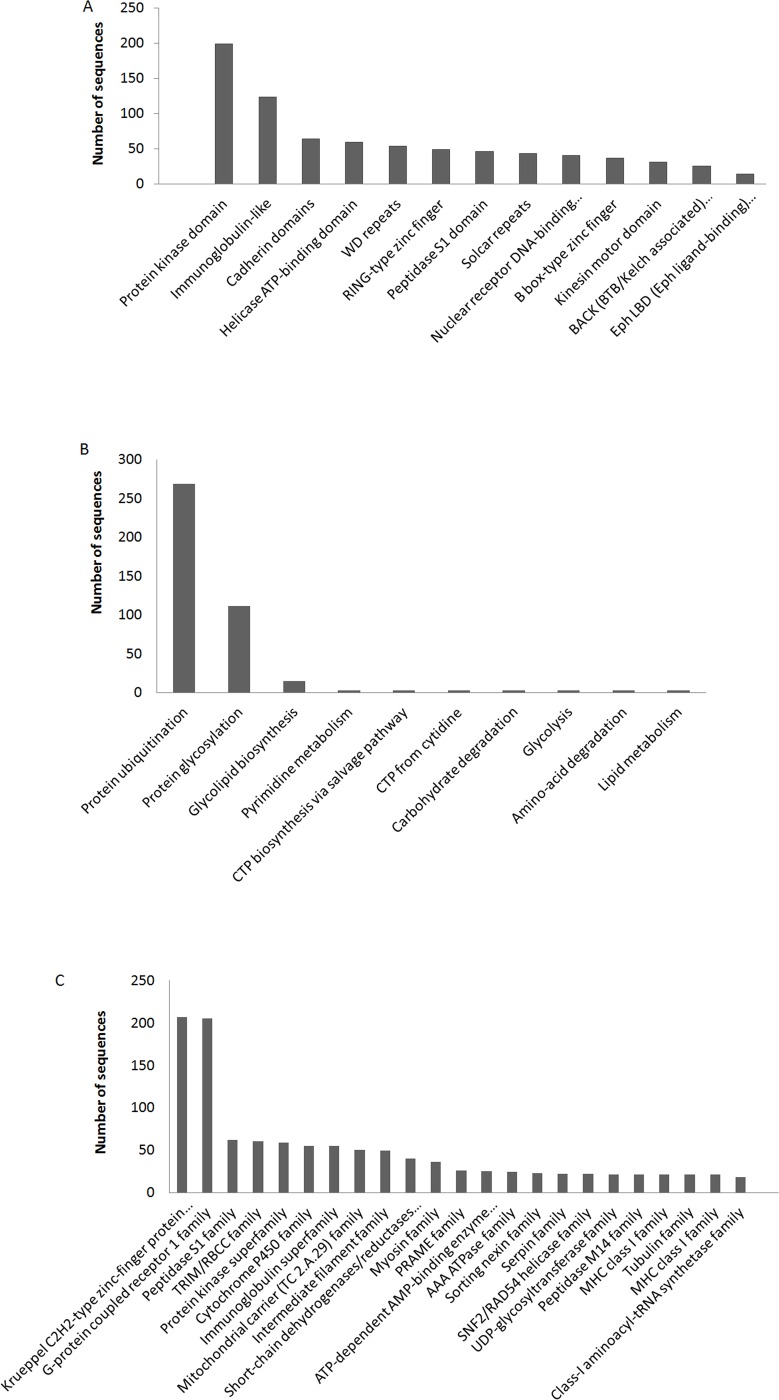
Function annotations of domain-swapped predicted sequences of human genome at three different levels. (A) Different Pfam protein families having maximum number of domain swapped predicted sequences from human genome. (B) Maximum number of protein sequences present in these biological pathways. (C) Distribution of these sequences in different protein families.

In the case of sequences positively predicted sequences by both RF and SVM methods (44%), a detailed analysis of the presence of domains was carried out and 2346 domain architectures were observed. Domain distributions of these sequences were also examined and it was observed that protein kinases, Immunoglobulin-like domain and cadherin domains are most populated (S2 Table). Other populated Pfam domain families in these sequences are shown in Fig 3A.

**Fig 4:**
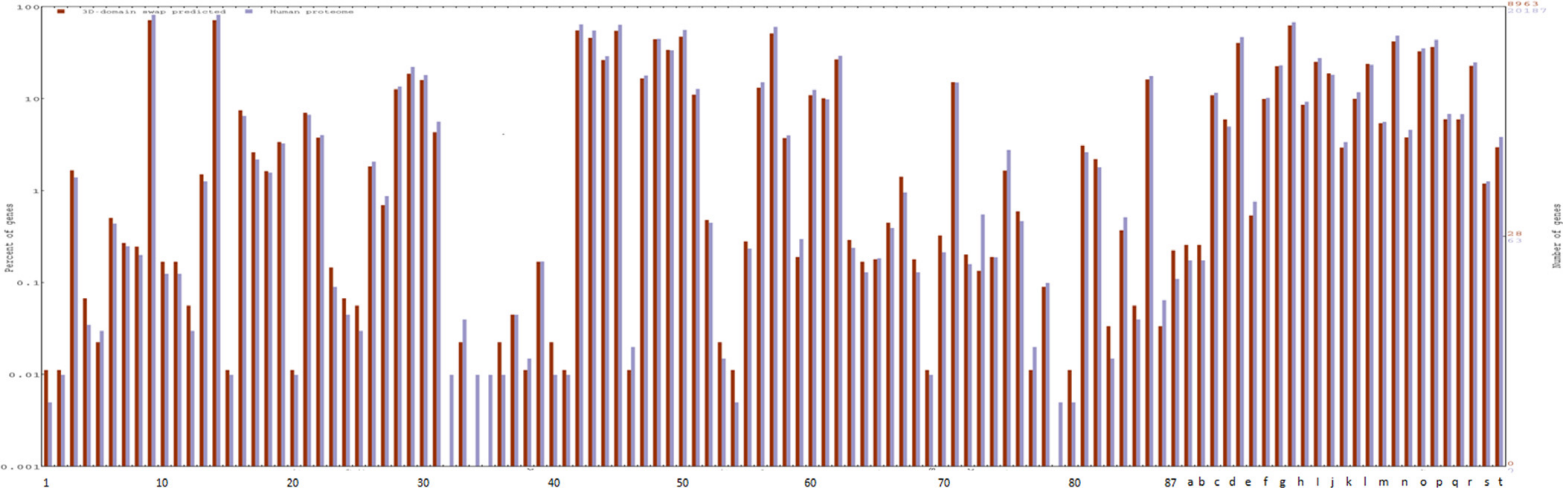
Preferred Gene Ontology (GO) terms in positively predicted sequences from human genome. Human genome is used as reference point and WEGO plotting tools is used. List of the GO terms as cellular component and biological functions, corresponding to X-axis labels, are provided in S1 File.

Further, these positively predicted sequences were enriched in 254 metabolic pathways. Protein ubiquitination and protein glycosylation pathways have a maximum number of sequences from 3D-domain swap predicted dataset (Fig 3B). Top enriched 3D-domain swap predicted sequences in different protein families were plotted (Fig 3C). Functional annotation and enrichment analysis was performed by using Gene Ontology terms for biochemical activity (Fig 4) and it resulted in 40 GO terms with fold enrichment ranging from 1.2 to 1.5 with significant E- values. The terms with maximum fold enrichment are cation channel activity (GO:0005261), metal ion transmembrane transporter activity (GO:0046873), gated channel activity (GO:0022836), ion channel activity (GO:0005216), ATPase activity (GO:0016887), adenyl nucleotide binding (GO:0030554), adenyl ribonucleotide binding (GO:0032559), ATP binding (GO:0005524), purine nucleoside binding (GO:0001883), nucleoside binding (GO:0001882), protein serine/threonine kinase (GO:0004674) and substrate-specific channel activity (GO:0022838).

For the disease association and biological pathway of these positively predicted sequences, Reactome database was also consulted. The major diseases associated with the predicted sequences are classified into 18 groups (Table 3), few of the most enriched groups based on Z-score are abnormal metabolism in phenylketonuria (0.33), defects in vitamin and cofactor metabolism (0.29), signaling by EGFR in cancer (0.26), disease associated with visual transduction (0.26), signaling in FGFR (0.25) etc. Surprisingly, Amyloidosis is not enriched in disease association section in this dataset and it may be since our dataset contains only reviewed entries from human proteome. Most of these entries are from signaling pathways leading to cancers. Other possibility is that domain swapping is not strongly associated with amyloidosis. All the biological pathways prevalent in this dataset are grouped into 34 biological pathways (Table 4). Some highly prevalent pathways based on Z-score are reproduction (0.34), neuronal system (0.30) and cell-cell communication (0.28). The full list of genes, enriched for Reactome terms are available at http://caps.ncbs.res.in/download/Human_pred/reactome/

**Table 3:** Disease association of 3D-domain swapped predicted protein sequences of human genome. Normalization (Z score) was calculated based on domain swapped entries, in whole human genome (8945/20247).

S. No.	Disease	Distribution	Z-score	FDR
1	HIV infection	109/229	0.21	1E^0^
2	Influenza infection	37/153	0.11	1E^0^
3	Latent infection of *Homo sapiens* with *Mycobacterium tuberculosis*	14/68	0.09	1E^0^
4	Uptake and actions of bacterial toxins	24/44	0.24	1E^0^
5	Signaling by EGFR in cancer	110/189	0.26	1E^0^
6	Signaling in FGFR	110/191	0.25	1E^0^
7	Abnormal metabolism in phenylketonuria	3/4	0.33	9.9E^-1^
8	Mucopolysaccharidosis	62/123	0.22	1E^0^
9	Disease associated with visual transduction	55/92	0.26	1E^0^
10	PI3K/AKT signaling in cancer	55/107	0.23	1E^0^
11	Signaling by NOTCH1 in cancer	39/73	0.24	1E^0^
12	Glycogen storage diseases	144/265	0.24	1E^0^
13	Defects in vitamin and cofactor metabolism	59/90	0.29	10E^-1^
14	Signaling by TGF-beta receptor complex in cancer	38/70	0.24	1E^0^
15	Disease of glycosylation	122/229	0.23	1E^0^
16	Signaling by WNT in cancer	87/212	0.18	1E^0^
17	Processing-defective Hh variants abrogate ligand secretion	28/64	0.19	1E^0^
18	Metabolic disorders of biological oxidation enzymes	317/616	0.23	1E^0^

**Table 4:** Distribution of genes in different biological pathways in positively predicted sequences of human proteome. Normalization (Z score) was calculated based on domain swapped entries in whole human genome (8945/20247).

S.No.	Pathway	Distribution	Normalized (Z) score	FDR
1	Binding and uptake of ligands by scavenger receptors	16/195	0.04	1E^0^
2	Cell cycle	267/525	0.22	1E^0^
3	Cell-cell communication	87/138	0.28	1E^0^
4	Cellular response to stress	87/236	0.16	1E^0^
5	Chromatin organization	94/208	0.20	1E^0^
6	Circadian Clock	21/55	0.17	1E^0^
7	Developmental biology	282/531	0.23	1E^0^
8	Disease	977/1,991	0.22	1E^0^
9	DNA repair	57/115	0.22	1E^0^
10	DNA replication	49/105	0.21	1E^0^
11	Extracellular matrix organization	137/271	0.22	1E^0^
12	Gene expression	495/1,196	0.18	1E^0^
13	Homeostasis	303/512	0.26	1E^0^
14	Immune system	665/1,451	0.20	1E^0^
15	Membrane trafficking	115/206	0.25	1E^0^
16	Metabolism	865/1,585	0.20	1E^0^
17	Metabolism of proteins	332/692	0.21	1E^0^
18	Muscle contraction	24/51	0.21	1E^0^
19	Neuronal system	194/280	0.30	1E^0^
20	Organelle biogenesis and maintenance	156/332	0.21	1E^0^
21	Programmed cell death	77/162	0.21	1E^0^
22	Reproduction	20/26	0.34	9.9E^-1^
23	Signal transduction	894/2,094	0.19	1E^0^
24	Transmembrane transport of small molecules	387/624	0.27	1E^0^

### Hinge region prediction

**Fig 5:**
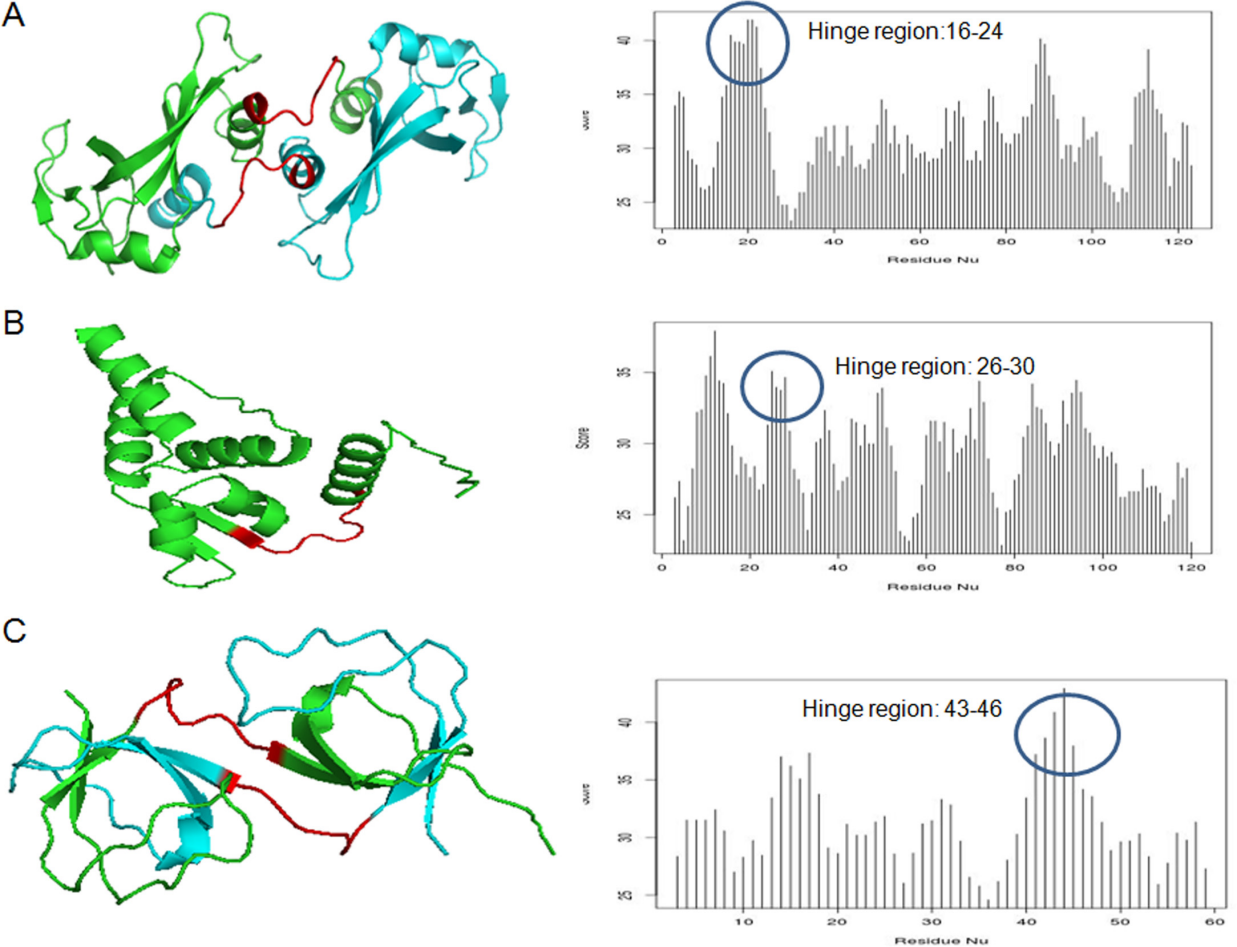
Case study on three different proteins of known structures. The blue circle shows that these are experimentally known hinge regions (shown in red in left) that agree with our predictions. (A) Ribonuclease, Seminal (PDB code: 11BA), (B) Promyelocytic leukemia Zinc finger protein PLZF (PDB code: 1BUO) and (C) SH3 Domain (PDB code: 1AOJ). Complete list is provided in S3 Table.

On the basis of experimentally reported hinge regions and swapped region of domain swapped protein structures, we found that the average hinge region is of 6.7 residues (ranging from one to 66 residues). A similar analysis was also performed on the length distribution of swapped domain in the known cases of domain swapping. Swapped domains are of an average size of 26 residues, ranging from 5 to 316 residues. Well-known examples of domain swapping were used for the study of hinge regions and were mapped back to structures as case study **(**Fig 5**).** Some of the case study examples chosen are seminal ribonuclease (11BA), promyelocytic leukemia Zinc finger protein PLZF (1BUO) and SH3 Domain (1AOJ).

Seminal ribonuclease retains an α+β fold, where the N-terminal region is engaged in 3D-domain swapping. This protein belongs to pancreatic ribonuclease domain family by Pfam database. Similarly, promyelocytic leukemia Zinc finger also belongs to α-and-β class and POZ fold according to SCOP database. It is a homodimer with N-terminal type of 3D-domain swapping. The normalized score (please see Methods for details) predicts the hinge region, as “NPSHPTGLLCK” (nineth to nineteenth residue of the protein sequence). These 11 residues at the N-terminal of the protein structure obtain maximum score and it also agrees with the experimentally demarcated hinge region in crystal structure. The prediction results were mapped on sequences based on cumulative maximum scores.

This prediction tool was applied to all the 3D-domain swapped predicted sequences (8945) of human genome. Prediction results of known examples of 3DSwap database were mapped on sequences based on cumulative maximum scores to continuous three residues. The regions with maximum score were called as first level of prediction. On the basis of these scores other putative hinges are also demarcated. For the first level, coverage of 67.8% was achieved, when the predicted best hinge was considered for calculation. When the second best hinge region on the protein sequence was also included, along with the best hinge region, a coverage of 71.3% was achieved.

### Challenges in hinge prediction at genome level

Although we have achieved a fairly good accuracy in the prediction of hinge region, there are many challenges which hinder this algorithm in achieving very good accuracy. Few of the challenges faced in this analysis are explained in this section. 3D-domain swapping mechanism was observed in almost all types of the proteins [11]. There was huge variability observed in number of subunits, position of swapped domain, type of swapped domain *etc*. in such molecules from one case to another. Besides these deviations, several other features arise, such as incomplete data (e.g. absence of coordinates of residues for significant region in protein structure), central loop swapped entries, small hinge region, domain swapped oligomer with elaborate interface, intertwined domain swapped proteins and multiple hinge regions pose severe challenges and were hard to address considering small frequency of occurrence. Small hinge regions, generally less than five residues, are difficult to predict since the cumulative scores could result in false positives (S1 Fig).

In case of central loop swapping, multiple hinge regions are involved and the swapped loop region exhibits majority of the characteristics of the hinge region. Hence, it is difficult to decide precise boundaries between swapped central loop and hinge region. In another problematic case, where intertwined structure is present, multiple hinge regions are present. Hence, it is almost impossible to identify hinge region in intertwined oligomers, even after manual observation by using visualization tools. These peculiar characteristics of the domain swapped molecules add to the difficulty of hinge prediction form mere sequence information of protein molecules. Besides, there is an inherent level of incomplete information, since the protein sequence space is much more vast than structural space.

### Disorder region prediction on the entire 3D-domain swap predicted sequences

There were only nine sequences predicted as completely disordered out of 11507 3D-domain swap predicted sequences by RF method. A total of nine percent (1040) of sequences have more than or equal to 50% disordered regions. The result shows that most of the sequences are well structured as found in globular proteins.

## Discussion and Conclusion

Early identification of 3D-domain swap proteins on genome scale would help in better management of disease caused by protein aggregation and other protein domain swap related phenomena. Machine learning approaches, classifiers (RF and SVM models) have been developed to predict domain swapping at the genome level from mere protein sequence information. An accuracy of 81% and 74% were achieved for the two methods, respectively. These prediction models were applied to several complete genomes with a special emphasis on the human genome. To our knowledge, this is the first attempt to predict domain swapping at the genome level. Almost 44% of the sequences of the human genome were predicted as putatively involved in domain swapping. We observed the same trend when we applied these two models to different genomes.

Functional annotation of all the positively predicted sequences from the human genome were carried out in terms of their distribution in different Pfam protein families, Gene Ontology, Biological pathways and Disease associations. These positively predicted sequences were dispersed in many Pfam protein families with different Gene Ontology annotations and in several pathways with substantial disease associations. Our approach helped us to understand enriched protein domains, Gene Ontology terms, biological pathways and Disease Ontology in putatively predicted protein sequences and their role in mediating various human diseases. Protein kinases, immunoglobulin-like domains and cadherin domains are most populated domain families observed in this dataset of sequences positively predicted for domain swapping. Few of the highly enriched human diseases in the positively-predicted dataset are, abnormal metabolism in phenylketonuria, defects in vitamin and cofactor metabolism, signaling by EGFR in cancer etc. The gene ontology terms for cation channel activity, metal ion transmembrane transporter activity, gated channel activity, ion channel activity, ATPase activity etc. have maximum fold enrichment under the biochemical activities.

We have also developed a hinge region prediction tool, mainly based on important physicochemical properties of the residues in the hinge region. Further distribution of these molecules across structure, sequence and function families shows generic nature of this mechanism emphasizing the fact that this mechanism occurs independent of sequence, structure and function.

Hinge region prediction was performed based on the important features of amino acids present in this region. This prediction tool is applied to all the predicted (8945) 3D-domain swapped sequences from human genome. Few of the peculiar features considered in this study are as (a) residues in the hinge region prefer to be small, but not hydrophobic or aliphatic in nature. (b) They are found less often in α-helices, and more often in turns or random coils. (c) Active site residues were found to coincide significantly with hinges. (d) Hinges are also more likely to occur on the protein surfaces than in core. (e) Propensity of amino acid residues in the hinges. (f) Hinge regions in domain swapped structures acquire distinguishing structural features.

Hinges retain extended loop conformation in the domain-swapped form and are always in proximity with the other subunit. Considering extended conformation and proximity to the other protomer, hinge residues forms very few or no intramolecular interactions but shows high degrees of intermolecular interactions. This characteristic of hinge residues is very crucial in deciding precise boundaries of hinge region, when structural coordinates are known.

The reliable prediction of which genes, and which regions of the genes, might be engaged in domain swapping could be useful for the design of bioengineering experiments and also to recognise potential target genes in various diseases.

## Materials and Methods

### Creation of positive and negative datasets

High quality domain-swapped dataset was obtained from 3DSwap+ database (manuscript under revision). In this updated version of the database, which is an automated and manually curated one, 2057 PDB entries of 3D-domain swapping protein entries are present with their sequence, structural and functional features.

**Fig 6:**
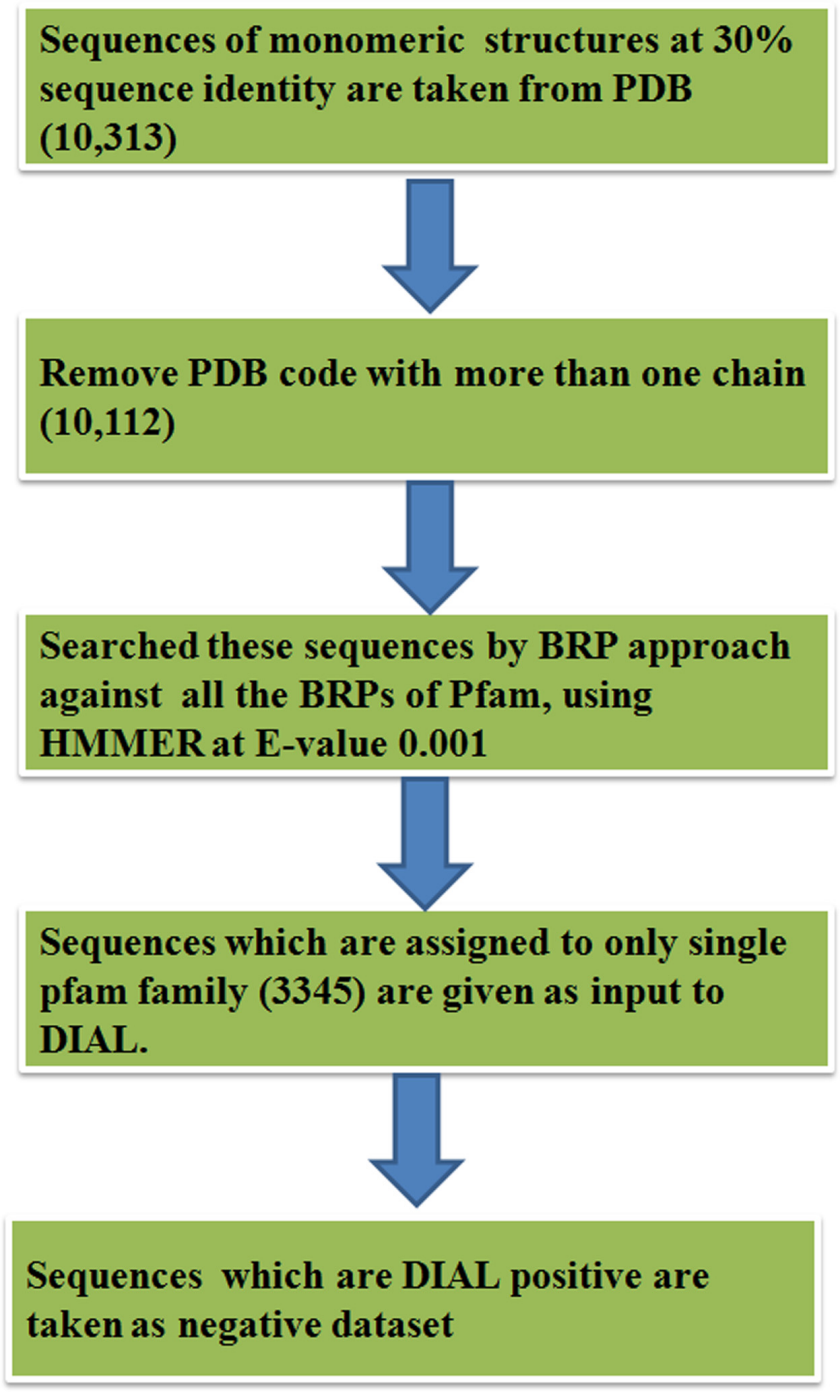
Workflow to generate negative dataset from monomeric structures of protein database (PDB). BRP approach was used to find the sequences form Pfam families which do not have known examples of domain swapping (please see Methods for details). DIAL was used for prediction of domain swapping in the given sequences.

For the creation of negative dataset, Best Representative Profile (BRP) approach was used, which assigns a best representative sequence (BRS) and also a BRP to a Pfam protein family [24]. All the monomeric structures (10,313) of PDB were taken at 30% sequence identity cut off. The 10,112 sequences of corresponding structures were searched against all the BRPs of Pfam [25] using HMMER [26], [27] at an E-value of 0.001. The sequences which were assigned to single Pfam protein family by BRP approach were provided as input to DIAL for the detection of structural domains in these sequences. The sequences which are made up of only single domain were assigned as negative dataset for domain swapping (Fig 6). There were 3200 sequences, assigned to only single domain by DIAL and used as negative dataset.

### Comparing positive and negative datasets

ProteinOrtho version 5.06, an orthology detection tool [28] was used to compare the positive and negative datasets. At default parameters, it was found that only 42 sequences (1%) have bidirectional edges out of 4000 sequences (2000 negative and positive sequences each). To check if the positive dataset (training dataset) has any bias towards the genome in which they are present, we have performed distribution analysis of these 2000 positive sequences in different taxonomic groups.

### Features used for model generation

A total of 439 sequence features was used for training the model. Out of 439 features, 39 were physicochemical features of amino acids extracted from AAINDEX database [29] and 400 were dipeptide features. These features were predicted as best features by WEKA software [30]. Features used in prediction model are listed in (Table 5).

**Table 5:** List of the features used for model generation for prediction of domain swapping.

S. No	Features	Number of feature
1	Propensity of amino acids in hinges	1
2	Hydrophobic index	1
3	Average flexibility indices	1
4	Residue volume	1
5	Transfer free energy surface	1
6	Normalized frequency of alpha helix	1
7	Normalized frequency of extended structure	1
8	Steric parameter	1
9	Polarizability parameter	1
10	Chou-Fasman parameter of coil conformation	1
11	Average volume of buried residue	1
12	Normalized frequency of beta turn	1
13	Normalized frequency of alpha helix	1
14	Normalized frequency of beta sheet	1
15	Normalized average of hydrophobicity scales	1
16	Partial specific volume	1
17	Normalized frequency of turn	1
18	Size	1
19	Relative mutability	1
20	Solvation free energy	1
21	Molecular weight	1
22	Positive charge	1
23	Negative charge	1
24	Composition	1
25	Polarity	1
26	Normalized relative frequency of extended structure	1
27	Average accessible surface area	1
28	Percentage buried residues	1
29	Percentage of exposed residues	1
30	Net charge	1
31	Normalized frequency of coil	1
32	Amino acid composition of total proteins	1
33	Optimized propensity to form reverse turn	1
34	Side chain orientational preference	1
35	Bulkiness	1
36	Isoelectric point	1
37	Normalized flexibility parameters	1
38	Amphiphilicity index	1
39	Linker propensity index	1
40	Composition based (Di-amino acid)	20*20 = 400
	Total features	439

### Work flow of model generation

**Fig 7:**
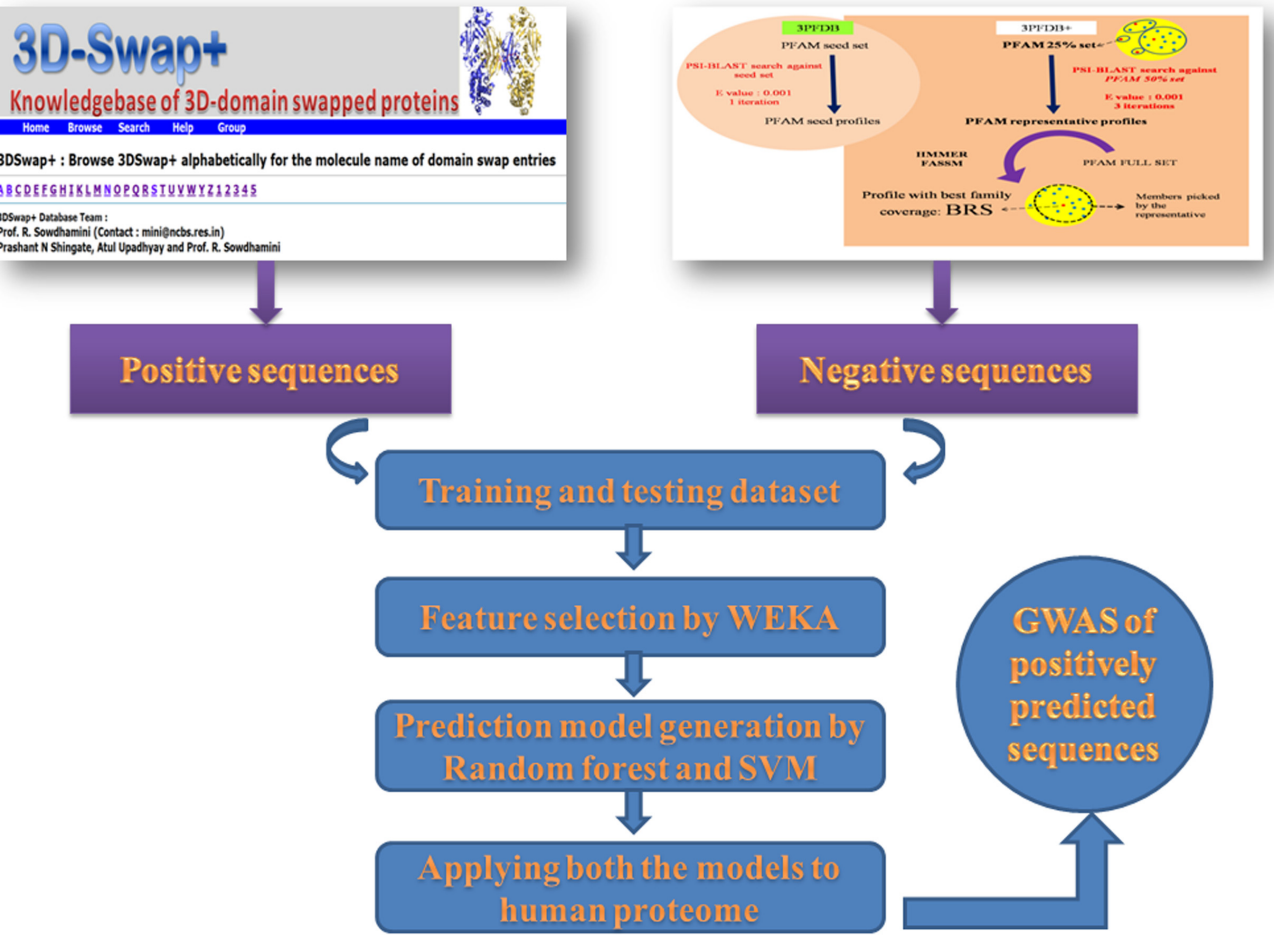
Overall workflow of the method used in this study and creation of positive and negative datasets. Feature selection by WEKA, and prediction model creation by Random Forest and Support Vector Machine. Genome-wide association study of sequences predicted to undergo domain swapping in the human genome.

For the generation of the prediction model, 2000 sequences from 3DSwap+ were used as positive dataset and 2000 sequences created by BRP approach were used as negative dataset. A Perl script was used to extract the numerical values based on AAINDEX of all the features of amino acid residues. All the numerical values of positive and negative sequences were saved in two comma separated values (csv) files. These two files were given as input to RF and SVM classifiers to generate models for the purpose of classification and a confusion matrix of 2*2 with True Positive (TP), True negative (TN), False Positive (FP) and False Negative (FN) values for training dataset was also generated (Fig 7). The confusion matrix for the testing datasets were calculated based on the prediction results.

### Five-fold Cross-validation of testing dataset

Evaluation of the prediction results was performed on a five-fold cross-validation method for the testing dataset. Cross-validation is a statistical technique for estimating the performance of a machine learning based prediction model. Fivefold cross-validation refers to an implementation of k-cross validation method (here *k = 5*). The original dataset is randomly divided into five derived datasets. Of these five datasets, a single one is retained as validation data for testing the classifier and remaining (5–1) derived datasets are used for training. The process is repeated five times (folds) with each of the derived datasets used only once as the validation data. These results from five-fold cross-validation datasets were averaged to obtain a final single estimation of statistical parameters.

### Statistical Assessment of the machine learning classifiers

The RF and SVM models were evaluated using few of important statistical assessment features viz., Accuracy, sensitivity, specificity and Mathew’s Correlation coefficient (MCC). These measurements are expressed in terms of the fraction of true positives (TP), false negatives (FN), true negatives (TN) and false positives (FP).

1. **Sensitivity.** Sensitivity refers to the percentage of sequences correctly predicted as “swapping” by the models.
  $$Sensitivity=\frac{TP}{TP+FN}$$
2. **Specificity.** Specificity refers to the percentage of sequences correctly predicted by the models as “non- swapping”.
  $$Specificity=\frac{TN}{TN+FP}$$
3. **Matthew’s correlation coefficient (MCC).** The MCC provides a combined measure of sensitivity and specificity. MCC ranges from –1 to 1. A value of MCC = 1 indicates the best correlation or possible prediction while MCC = -1 indicates the worst possible prediction or anti- correlation. Finally, MCC = 0 would be expected for a random prediction scheme.
  $$MCC=\frac{\left(TP*TN)-(FP*FN\right)}{\surd \{(TN+FN)(TP+FN)(TN+FP)(TP+FP)\}}$$
4. **Accuracy.** Accuracy refers to the percentage of correct prediction out of the total number of predictions.
  $$Accuracy=\frac{\left(TP+TN\right)}{\left(TP+FP+TN+FN\right)}$$

### Applying the model for prediction well-known cases of 3DSswap database and aggregation related proteins from human proteome

The RF and SVM models were applied to 137 reviewed, aggregation-related protein sequences from human proteome. These proteins are associated with different diseases such as amyloid forming proteins. The positively predicted 3D-domain swapping sequences were searched against PDB for their structural homologous at an E-value of 0.001.

### Applying RF and SVM models to few other genomes of animal and plant kingdom

Prediction models were applied on different genomes like *Mycobacterium tuberculosis* (Mtb), *Homo sapiens* and *Bacillus thuriengenesis* (Bth) and also on few plant genomes as *Solanum tuberosum* (Stu), *Ocimum tenuiflorum* (Ote), *Medicago sativa* (Msa) and *Arabidopsis thaliana* (Ath). All the protein sequences of these genomes were downloaded from UNIPROT.

### Proteome-wide prediction of human 3D-domain swapping

All the 20247 reviewed entries of the human genome were downloaded from UNIPROT to predict 3D-domain swapping at the complete proteome level (S3 Table). For these sequences, numerical values of each feature were extracted and the RF and SVM models were run against these values.

### Enrichment analysis of 3D-domain swapped predicted proteins from human proteome

Detailed analysis of these 3D-domain swapped predicted protein sequences (S4 Table) from human proteome was performed at different levels. Firstly, we studied disease association of these sequences based on information provided on UniProtROT database by mapping the accession codes of these sequences to disease association. The enrichment analysis of protein domains for these sequences were analyzed for Pfam domain information by using DAVID tool [31]. We also studied their functional importance and involvement in different biological processes from their Gene Ontology (GO) information by using DAVID tool [31]. Detailed analysis of these sequences in terms of pathways and biological processes was performed using REACTOME database [32], which is an open-source, open-access, manually curated, peer-reviewed database of human pathways and processes. These annotations were plotted by using online server WEGO [33].

### Disorder region prediction on the entire 3D-domain swap predicted sequences

Espritz–an efficient online server for detection of protein disorder [34] was applied on all the 3D-domain predicted sequences from human proteome using default parameters.

### Hinge region prediction from protein sequences of 3D-domain swapped sequences

Hinge region prediction has been performed in the 3D-domain swapped predicted sequences by using a novel algorithm based on important features. The distribution of hinge region and swapped domain lengths were analyzed for the known examples of 3D-domain-swapped protein structures. On the basis of analysis done on the known examples of 3D-domain swapped molecules of 3DSwap+ database (~2000 entries), a minimum hinge size of three residues and maximum of seven residues were found. Six physicochemical features form hinge region sequences were employed in this algorithm. These features are (1) Propensity of amino acids in the hinges [21], (2) Average flexibility index [35], (3) Normalized frequency of extended structure [36], (4) Normalized frequency of coil [37], (5) Normalized frequency of beta turn [38], and (6) Relative mutability [39]. In this algorithm, we have used a sliding window approach of five and a score is assigned to all the residues starting from third residue to last-but-third residues. These scores are plotted against the residue number by an automated Perl script, which is called into R prompt. The average best score of the consecutive three residues is taken into consideration. There were five best hinge regions marked in the result file based on the average best score of three consecutive residues.

The normalization of the score was done by following formula:

$$N=\frac{S-Y}{X-Y}$$

Where,

N = Normalized score of each residue

S = Cumulative score of a residue in the sequence

X = Maximum score in the sequence

Y = Maximum score of any residue in the given sequence

### Performance of hinge prediction tool

The coverage of the tool was calculated using following formula:

$$C=\frac{Rh}{Rh+W}$$

Where,

C = Coverage

Rh = Number of proteins in, which whole or part of identified hinge region overlaps with reference hinge

W = Number of proteins in, which, identified hinge and reference hinge are totally different

## Supporting Information

S1 Fig **Examples of challenging cases for hinge prediction, experimentally identified hinges are marked in blue color** (A: Central loop swapping (PDB: 1IXX), B and C: Intertwined dimmers (PDB: 1A64), D: Missing regions (PDB: 2ZEJ), E: Very small hinges (PDB: 5CRO) and F: Elaborate interface (2PJW))
(TIF)

S1 File Description of Gene Ontology terms prevalent in human domain swap predicted sequences. (DOC)

S1 Table Prediction results on known cases of domain swapping from 3DSwap as validation. (DOC)

S2 Table Predicted 3D-domain swapped human entries with Pfam domain family associations. (TXT)

S3 Table Reviewed UniProt sequences of human proteome used as input to prediction models. (TXT)

S4 Table Predicted 3D-domain swapped sequences of human proteome. (TXT)
