# Supplementary figures and images for: Genome-Wide Prediction and Analysis of 3D-Domain Swapped Proteins in the Human Genome from Sequence Information
Source: PLoS One. 2016 Jul 28;11(7):e0159627. doi: 10.1371/journal.pone.0159627 (PMC4965083; doi:10.1371/journal.pone.0159627)

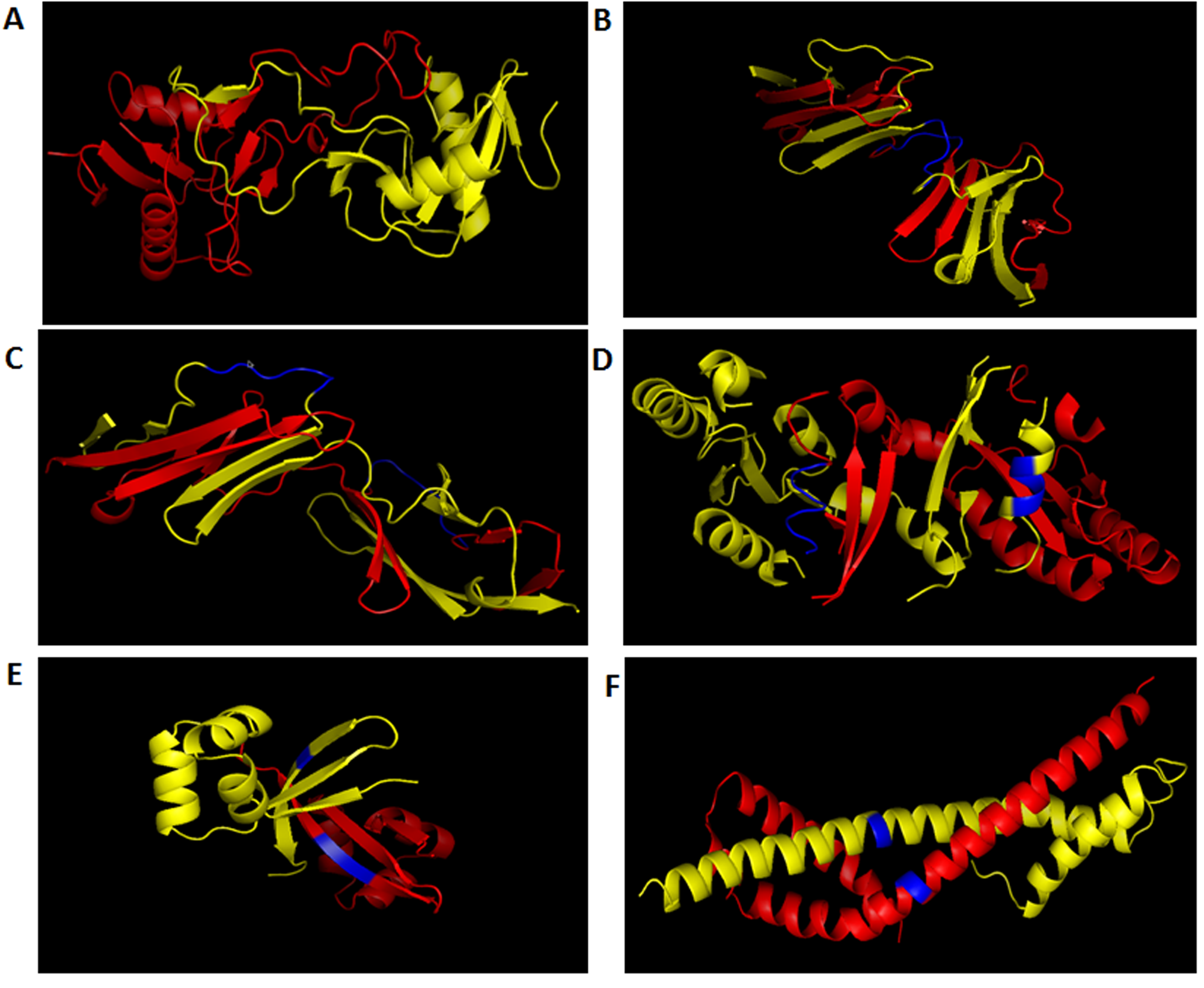

Supplement: S1 Fig — Examples of challenging cases for hinge prediction, experimentally identified hinges are marked in blue color (A: Central loop swapping (PDB: 1IXX), B and C: Intertwined dimmers (PDB: 1A64), D: Missing regions (PDB: 2ZEJ), E: Very small hinges (PDB: 5CRO) and F: Elaborate interface (2PJW)) (TIF) [file pone.0159627.s001.tif]
