# Supplementary material for: Genome-Wide Prediction and Analysis of 3D-Domain Swapped Proteins in the Human Genome from Sequence Information
Source: PLoS One. 2016 Jul 28;11(7):e0159627. doi: 10.1371/journal.pone.0159627 (PMC4965083; doi:10.1371/journal.pone.0159627)
Supplement: S1 File — (DOC) [file pone.0159627.s002.doc]

**S1 file:** Description of Gene Ontology terms prevalent in human domain swap predicted sequences.

**Cellular component**

1. Activin complex
2. Angiogenin –PRI complex
3. Apical part cell
4. Asymmetric synapse
5. Axon hillock
6. Axoneme
7. Axoneme part
8. Basal part of cell
9. Cell
10. Cell division site
11. Cell division site part
12. Cell envelop
13. Cell leading edge
14. Cell part
15. Cell pole
16. Cell projection
17. Cell projection part
18. Cell soma
19. Cell surface
20. Complement component C1 complex
21. Endomembrane system
22. Envelop
23. Excitatory synapse
24. External encapsulating structures
25. External encapsulating structures part
26. Extracellular matrix
27. Extracellular matrix part
28. Extracellular organelle
29. Extracellular region
30. Extracellular region part
31. Extracellular space
32. Fibrinogen complex
33. Host cell
34. Host cell part
35. Inhibin complex
36. Inhibitory complex
37. Insulin-like growth factor binding protein complex
38. Intercellular bridge
39. Interleukin-12 complex
40. Interleukin-23 complex
41. Intracellular
42. Intracellular organelle
43. Intracellular organelle part
44. Intracellular part
45. Kinocilium
46. Macromolecular complex
47. Membrane
48. Membrane part
49. Membrane-bound organelle
50. Membrane-enclosed lumen
51. Midbody
52. Molybdopterin synthase complex
53. Mucus layer
54. Neuromuscular junction
55. Non-membrane-bounded organelle
56. Organelle
57. Organelle envelope
58. Organelle envelope lumen
59. Organelle lumen
60. Organelle membrane
61. Organelle part
62. Perikaryon
63. Photoreceptor inner segment
64. Plasma lipoprotein particle
65. Postsynaptic density
66. Postsynaptic active zone
67. Presynaptic cytoskeletal matrix assembled at active zone
68. Presynaptic membrane
69. Protein complex
70. Protein serine/threonine phosphatase complex
71. Protein-DNA complex
72. Protein-lipid complex
73. Ribonucleoprotein complex
74. Site of polarized growth
75. Stereocilia coupling link
76. Stereocilium membrane
77. Symmetric synapse
78. Synapse
79. Synapse part
80. Synaptic cleft
81. Synaptic vesicle
82. Trailing edge
83. Vesicle
84. Viral capsid
85. Viral envelop
86. Virion
87. Virion part

**Biological process**

1. Anatomical structure formation
2. Biological adhesion
3. Biological regulation
4. Cell killing
5. Cellular component biogenesis
6. Cellular component organization
7. Cellular process
8. Establishment of localization
9. Growth
10. Immune system process
11. Localization
12. Metabolic process
13. Multi-organ process
14. Multicellular organismal process
15. Pigmentation
16. Reproduction
17. Reproductive process
18. Response to stimulus
19. Rhythmic process
20. Viral reproduction
