# Supplementary material for: Genome-Wide Prediction and Analysis of 3D-Domain Swapped Proteins in the Human Genome from Sequence Information
Source: PLoS One. 2016 Jul 28;11(7):e0159627. doi: 10.1371/journal.pone.0159627 (PMC4965083; doi:10.1371/journal.pone.0159627)
Supplement: S1 Table — (DOC) [file pone.0159627.s003.doc]

S1 Table: Prediction results on known cases of domain swapping from 3DSwap as validation

| S. No. | PDB | Protein Name | Resolution | Prediction results by Random Forest | Prediction results by SVM |
| --- | --- | --- | --- | --- | --- |
| 1 | 11BA | BINDING OF A SUBSTRATE ANALOGUE TO A DOMAIN SWAPPING PROTEIN IN THE COMPLEX OF BOVINE SEMINAL RIBONUCLEASE WITH URIDYLYL-2',5'-ADENOSINE | 2.06 | yes | yes |
| 2 | 11BG | A POTENTIAL ALLOSTERIC SUBSITE GENERATED BY DOMAIN SWAPPING IN BOVINE SEMINAL RIBONUCLEASE | 1.9 | yes | yes |
| 3 | 1A2W | CRYSTAL STRUCTURE OF A 3D DOMAIN-SWAPPED DIMER OF BOVINE PANCREATIC RIBONUCLEASE A | 2.1 | yes | yes |
| 4 | 1A64 | ENGINEERING A MISFOLDED FORM OF RAT CD2 | 2 | yes | yes |
| 5 | 1AOJ | THE SH3 DOMAIN OF EPS8 EXISTS AS A NOVEL INTERTWINED DIMER | 2.5 | yes | yes |
| 6 | 1BH5 | HUMAN GLYOXALASE I Q33E,E172Q DOUBLE MUTANT | 2.2 | yes | yes |
| 7 | 1BJ3 | CRYSTAL STRUCTURE OF COAGULATION FACTOR IX-BINDING PROTEIN (IX-BP) FROM VENOM OF HABU SNAKE WITH A HETERODIMER OF C-TYPE LECTIN DOMAINS | 2.6 | yes | yes |
| 8 | 1BL9 | CONFORMATIONAL CHANGES OCCURRING UPON REDUCTION IN NITRITE REDUCTASE FROM PSEUDOMONAS AERUGINOSA | 2.9 | yes | yes |
| 9 | 1BLB | CLOSE PACKING OF AN OLIGOMERIC EYE LENS BETA-CRYSTALLIN INDUCES LOSS OF SYMMETRY AND ORDERING OF SEQUENCE EXTENSIONS | 3.3 | yes | yes |
| 10 | 1BSR | BOVINE SEMINAL RIBONUCLEASE STRUCTURE AT 1.9 ANGSTROMS RESOLUTION | 1.9 | yes | yes |
| 11 | 1BUO | BTB DOMAIN FROM PLZF | 1.9 | yes | yes |
| 12 | 1BYL | BLEOMYCIN RESISTANCE PROTEIN FROM STREPTOALLOTEICHUS HINDUSTANUS | 2.3 | yes | yes |
| 13 | 1CDC | CD2, N-TERMINAL DOMAIN (1-99), TRUNCATED FORM | 2 | yes | yes |
| 14 | 1CKS | HUMAN CKSHS2 ATOMIC STRUCTURE: A ROLE FOR ITS HEXAMERIC ASSEMBLY IN CELL CYCLE CONTROL | 2.1 | yes | yes |
| 15 | 1CQZ | CRYSTAL STRUCTURE OF MURINE SOLUBLE EPOXIDE HYDROLASE. | 2.8 | yes | yes |
| 16 | 1CR6 | CRYSTAL STRUCTURE OF MURINE SOLUBLE EPOXIDE HYDROLASE COMPLEXED WITH CPU INHIBITOR | 2.8 | yes | yes |
| 17 | 1CTS | CRYSTALLOGRAPHIC REFINEMENT AND ATOMIC MODELS OF TWO DIFFERENT FORMS OF CITRATE SYNTHASE AT 2.7 AND 1.7 ANGSTROMS RESOLUTION | 2.7 | yes | yes |
| 18 | 1DDT | THE REFINED STRUCTURE OF DIMERIC DIPHTHERIA TOXIN AT 2.0 ANGSTROMS RESOLUTION | 2 | yes | yes |
| 19 | 1DUD | DEOXYURIDINE 5'-TRIPHOSPHATE NUCLEOTIDE HYDROLASE (D-UTPASE) COMPLEXED WITH THE SUBSTRATE ANALOGUE DEOXYURIDINE 5'-DIPHOSPHATE (D-UDP) | 2.3 | yes | yes |
| 20 | 1DWW | MURINE INDUCIBLE NITRIC OXIDE SYNTHASE OXYGENASE DIMER N-HYDROXYARGININE AND DIHYDROBIOPTERIN | 2.35 | yes | yes |
| 21 | 1DXX | N-TERMINAL ACTIN-BINDING DOMAIN OF HUMAN DYSTROPHIN | 2.6 | yes | yes |
| 22 | 1DZ3 | DOMAIN-SWAPPING IN THE SPORULATION RESPONSE REGULATOR SPO0A | 1.65 | yes | yes |
| 23 | 1E0J | GP4D HELICASE FROM PHAGE T7 ADPNP COMPLEX | 3 | yes | yes |
| 24 | 1E7D | ENDONUCLEASE VII (ENDOVII) FROM PHAGE T4 | 2.8 | yes | yes |
| 25 | 1E7L | ENDONUCLEASE VII (ENDOVII) N62D MUTANT FROM PHAGE T4 | 1.32 | yes | yes |
| 26 | 1EGG | STRUCTURE OF A C-TYPE CARBOHYDRATE-RECOGNITION DOMAIN (CRD-4) FROM THE MACROPHAGE MANNOSE RECEPTOR | 2.3 | yes | yes |
| 27 | 1EGI | STRUCTURE OF A C-TYPE CARBOHYDRATE-RECOGNITION DOMAIN (CRD-4) FROM THE MACROPHAGE MANNOSE RECEPTOR | 2.3 | yes | yes |
| 28 | 1EK1 | CRYSTAL STRUCTURE OF MURINE SOLUBLE EPOXIDE HYDROLASE COMPLEXED WITH CIU INHIBITOR | 3.1 | yes | yes |
| 29 | 1EK2 | CRYSTAL STRUCTURE OF MURINE SOLUBLE EPOXIDE HYDROLASE COMPLEXED WITH CDU INHIBITOR | 3 | yes | yes |
| 30 | 1EN7 | ENDONUCLEASE VII (ENDOVII) FROM PHAGE T4 | 2.4 | yes | yes |
| 31 | 1F0V | Crystal structure of an Rnase A dimer displaying a new type of 3D domain swapping | 1.7 | yes | yes |
| 32 | 1F2N | RICE YELLOW MOTTLE VIRUS | 2.8 | yes | yes |
| 33 | 1F61 | CRYSTAL STRUCTURE OF ISOCITRATE LYASE FROM MYCOBACTERIUM TUBERCULOSIS | 2 | yes | yes |
| 34 | 1F8I | CRYSTAL STRUCTURE OF ISOCITRATE LYASE:NITROPROPIONATE:GLYOXYLATE COMPLEX FROM MYCOBACTERIUM TUBERCULOSIS | 2.25 | yes | yes |
| 35 | 1F8M | CRYSTAL STRUCTURE OF 3-BROMOPYRUVATE MODIFIED ISOCITRATE LYASE (ICL) FROM MYCOBACTERIUM TUBERCULOSIS | 1.8 | yes | yes |
| 36 | 1FLO | FLP Recombinase-Holliday Junction Complex I | 2.65 | yes | yes |
| 37 | 1FRO | HUMAN GLYOXALASE I WITH BENZYL-GLUTATHIONE INHIBITOR | 2.2 | yes | yes |
| 38 | 1FYR | DIMER FORMATION THROUGH DOMAIN SWAPPING IN THE CRYSTAL STRUCTURE OF THE GRB2-SH2 AC-PYVNV COMPLEX | 2.4 | yes | yes |
| 39 | 1FZR | CRYSTAL STRUCTURE OF BACTERIOPHAGE T7 ENDONUCLEASE I | 2.1 | yes | yes |
| 40 | 1G5C | CRYSTAL STRUCTURE OF THE 'CAB' TYPE BETA CLASS CARBONIC ANHYDRASE FROM METHANOBACTERIUM THERMOAUTOTROPHICUM | 2.1 | yes | yes |
| 41 | 1G6U | CRYSTAL STRUCTURE OF A DOMAIN SWAPPED DIMER | 1.48 | yes | yes |
| 42 | 1G85 | CRYSTAL STRUCTURE OF BOVINE ODORANT BINDING PROTEIN COMPLEXED WITH IS NATURAL LIGAND | 1.8 | yes | yes |
| 43 | 1G96 | HUMAN CYSTATIN C; DIMERIC FORM WITH 3D DOMAIN SWAPPING | 2.5 | yes | yes |
| 44 | 1GME | CRYSTAL STRUCTURE AND ASSEMBLY OF AN EUKARYOTIC SMALL HEAT SHOCK PROTEIN | 2.7 | yes | yes |
| 45 | 1GP9 | A NEW CRYSTAL FORM OF THE NK1 SPLICE VARIANT OF HGF/SF DEMONSTRATES EXTENSIVE HINGE MOVEMENT AND SUGGESTS THAT THE NK1 DIMER ORIGINATES BY DOMAIN SWAPPING | 2.5 | yes | yes |
| 46 | 1GT1 | COMPLEX OF BOVINE ODORANT BINDING PROTEIN WITH AMINOANTHRACENE AND PYRAZINE | 1.71 | yes | yes |
| 47 | 1GT3 | COMPLEX OF BOVINE ODORANT BINDING PROTEIN WITH DIHYDROMYRCENOL | 1.8 | yes | yes |
| 48 | 1GT4 | COMPLEX OF BOVINE ODORANT BINDING PROTEIN WITH UNDECANAL | 2.1 | yes | yes |
| 49 | 1GT5 | COMPLEXE OF BOVINE ODORANT BINDING PROTEIN WITH BENZOPHENONE | 2.08 | yes | yes |
| 50 | 1H8X | DOMAIN-SWAPPED DIMER OF A HUMAN PANCREATIC RIBONUCLEASE VARIANT | 2 | yes | yes |
| 51 | 1HE7 | HUMAN NERVE GROWTH FACTOR RECEPTOR TRKA | 2 | yes | yes |
| 52 | 1HT9 | DOMAIN SWAPPING EF-HANDS | 1.76 | yes | yes |
| 53 | 1HUL | A NOVEL DIMER CONFIGURATION REVEALED BY THE CRYSTAL STRUCTURE AT 2.4 ANGSTROMS RESOLUTION OF HUMAN INTERLEUKIN-5 | 2.4 | yes | yes |
| 54 | 1HW7 | HSP33, HEAT SHOCK PROTEIN WITH REDOX-REGULATED CHAPERONE ACTIVITY | 2.2 | yes | yes |
| 55 | 1I1D | CRYSTAL STRUCTURE OF YEAST GNA1 BOUND TO COA AND GLNAC-6P | 1.8 | yes | yes |
| 56 | 1I21 | CRYSTAL STRUCTURE OF YEAST GNA1 | 2.4 | yes | yes |
| 57 | 1I4M | Crystal structure of the human prion protein reveals a mechanism for oligomerization | 2 | yes | yes |
| 58 | 1IIP | Bovine Cyclophilin 40, Tetragonal Form | 2 | yes | yes |
| 59 | 1ILK | INTERLEUKIN-10 CRYSTAL STRUCTURE REVEALS THE FUNCTIONAL DIMER WITH AN UNEXPECTED TOPOLOGICAL SIMILARITY TO INTERFERON GAMMA | 1.8 | yes | yes |
| 60 | 1IOD | CRYSTAL STRUCTURE OF THE COMPLEX BETWEEN THE COAGULATION FACTOR X BINDING PROTEIN FROM SNAKE VENOM AND THE GLA DOMAIN OF FACTOR X | 2.3 | yes | yes |
| 61 | 1IXX | CRYSTAL STRUCTURE OF COAGULATION FACTORS IX/X-BINDING PROTEIN (IX/X-BP) FROM VENOM OF HABU SNAKE WITH A HETERODIMER OF C-TYPE LECTIN DOMAINS | 2.5 | yes | yes |
| 62 | 1J30 | The crystal structure of sulerythrin, a rubrerythrin-like protein from a strictly aerobic and thermoacidiphilic archaeon | 1.7 | yes | yes |
| 64 | 1JML | Conversion of Monomeric Protein L to an Obligate Dimer by Computational Protein Design | 1.9 | yes | yes |
| 65 | 1JS0 | Crystal Structure of 3D Domain-swapped RNase A Minor Trimer | 2.2 | yes | yes |
| 66 | 1JWI | Crystal Structure of Bitiscetin, a von Willeband Factor-dependent Platelet Aggregation Inducer. | 2 | yes | yes |
| 67 | 1JYU | Xray Structure of Grb2 SH2 Domain | 2.75 | yes | yes |
| 68 | 1K04 | Crystal Structure of the Focal Adhesion Targeting Domain of Focal Adhesion Kinase | 1.95 | yes | yes |
| 69 | 1K46 | Crystal Structure of the Type III Secretory Domain of Yersinia YopH Reveals a Domain-Swapped Dimer | 2.2 | yes | yes |
| 70 | 1K4Z | C-terminal Domain of Cyclase Associated Protein | 2.3 | yes | yes |
| 71 | 1K50 | A V49A Mutation Induces 3D Domain Swapping in the B1 Domain of Protein L from Peptostreptococcus magnus | 1.8 | yes | yes |
| 72 | 1K51 | A G55A Mutation Induces 3D Domain Swapping in the B1 Domain of Protein L from Peptostreptococcus magnus | 1.8 | yes | yes |
| 73 | 1K6W | The Structure of Escherichia coli Cytosine Deaminase | 1.75 | yes | yes |
| 74 | 1K70 | The Structure of Escherichia coli Cytosine Deaminase bound to 4-Hydroxy-3, 4-Dihydro-1H-Pyrimidin-2-one | 1.8 | yes | yes |
| 75 | 1K75 | The L-histidinol dehydrogenase (hisD) structure implicates domain swapping and gene duplication. | 1.75 | yes | yes |
| 76 | 1K9U | Crystal Structure of the Calcium-Binding Pollen Allergen Phl p 7 (Polcalcin) at 1.75 Angstroem | 1.75 | yes | yes |
| 77 | 1KAE | L-HISTIDINOL DEHYDROGENASE (HISD) STRUCTURE COMPLEXED WITH L-HISTIDINOL (SUBSTRATE), ZINC AND NAD (COFACTOR) | 1.7 | yes | yes |
| 78 | 1KAH | L-HISTIDINOL DEHYDROGENASE (HISD) STRUCTURE COMPLEXED WITH L-HISTIDINE (PRODUCT), ZN AND NAD (COFACTOR) | 2.1 | yes | yes |
| 79 | 1KAR | L-HISTIDINOL DEHYDROGENASE (HISD) STRUCTURE COMPLEXED WITH HISTAMINE (INHIBITOR), ZINC AND NAD (COFACTOR) | 2.1 | yes | yes |
| 80 | 1KLL | Molecular basis of mitomycin C resictance in streptomyces: Crystal structures of the MRD protein with and without a drug derivative | 1.5 | yes | yes |
| 81 | 1L5B | DOMAIN-SWAPPED CYANOVIRIN-N DIMER | 2 | yes | yes |
| 83 | 1L5X | The 2.0-Angstrom resolution crystal structure of a survival protein E (SurE) homolog from Pyrobaculum aerophilum | 2 | yes | yes |
| 84 | 1L6W | Fructose-6-phosphate aldolase | 1.93 | yes | yes |
| 85 | 1LGP | Crystal structure of the FHA domain of the Chfr mitotic checkpoint protein complexed with tungstate | 2 | yes | yes |
| 86 | 1LGQ | Crystal structure of the FHA domain of the Chfr mitotic checkpoint protein | 2.1 | yes | yes |
| 87 | 1LOM | CYANOVIRIN-N DOUBLE MUTANT P51S S52P | 1.72 | yes | yes |
| 88 | 1LSS | KTN Mja218 CRYSTAL STRUCTURE IN COMPLEX WITH NAD+ | 2.3 | yes | yes |
| 89 | 1LSU | KTN Bsu222 Crystal Structure in Complex with NADH | 2.85 | yes | yes |
| 90 | 1LXE | CRYSTAL STRUCTURE OF THE CATHELICIDIN MOTIF OF PROTEGRINS | 2.5 | yes | yes |
| 91 | 1M0D | Crystal Structure of Bacteriophage T7 Endonuclease I with a Wild-Type Active Site and Bound Manganese Ions | 1.9 | yes | yes |
| 92 | 1M0I | Crystal Structure of Bacteriophage T7 Endonuclease I with a Wild-Type Active Site | 2.55 | yes | yes |
| 93 | 1M3D | Structure of Type IV Collagen NC1 Domains | 2 | yes | yes |
| 94 | 1MFZ | Partially refined 2.8 A Crystal structure of GDP-mannose dehydrogenase from P. aeruginosa | 2.8 | yes | yes |
| 95 | 1MI7 | Crystal Structure of Domain Swapped trp Aporepressor in 30%(v/v) Isopropanol | 2.5 | yes | yes |
| 96 | 1MN3 | Cue domain of yeast Vps9p | 2.3 | yes | yes |
| 97 | 1MO1 | CRYSTAL STRUCTURE AT 1.8 ANGSTROMS OF SELENO METHIONYLED CRH, THE BACILLUS SUBTILIS CATABOLITE REPRESSION CONTAINING PROTEIN CRH REVEALS AN UNEXPECTED SWAPPING DOMAIN AS AN UNTERTWINNED DIMER | 1.8 | yes | yes |
| 98 | 1MU4 | CRYSTAL STRUCTURE AT 1.8 ANGSTROMS OF THE BACILLUS SUBTILIS CATABOLITE REPRESSION HISTIDINE CONTAINING PROTEIN (CRH) | 1.8 | yes | yes |
| 99 | 1MUU | 2.0 A crystal structure of GDP-mannose dehydrogenase | 2.02 | yes | yes |
| 100 | 1MV8 | 1.55 A crystal structure of a ternary complex of GDP-mannose dehydrogenase from Psuedomonas aeruginosa | 1.55 | yes | yes |
| 101 | 1N1C | Crystal Structure Of The Dimeric TorD Chaperone From Shewanella Massilia | 2.4 | yes | yes |
| 103 | 1NIR | OXYDIZED NITRITE REDUCTASE FROM PSEUDOMONAS AERUGINOSA | 2.15 | yes | yes |
| 104 | 1NNO | CONFORMATIONAL CHANGES OCCURRING UPON NO BINDING IN NITRITE REDUCTASE FROM PSEUDOMONAS AERUGINOSA | 2.65 | yes | yes |
| 105 | 1NNQ | rubrerythrin from Pyrococcus furiosus Pfu-1210814 | 2.35 | yes | yes |
| 106 | 1NPB | Crystal structure of the fosfomycin resistance protein from transposon Tn2921 | 2.5 | yes | yes |
| 107 | 1NPR | CRYSTAL STRUCTURE OF AQUIFEX AEOLICUS NUSG IN C222(1) | 2.21 | yes | yes |
| 108 | 1NQB | TRIVALENT ANTIBODY FRAGMENT | 2 | yes | yes |
| 109 | 1O4W | Crystal structure of PIN (PilT N-terminus) domain (AF0591) from Archaeoglobus fulgidus at 1.90 A resolution | 1.9 | yes | yes |
| 110 | 1O5O | Crystal structure of Uracil phosphoribosyltransferase (TM0721) from Thermotoga maritima at 2.30 A resolution | 2.3 | yes | yes |
| 111 | 1O5Q | Crystal Structure of Pyruvate and Mg2+ bound 2-methylisocitrate lyase (PrpB) from Salmonella typhimurium | 2.3 | yes | yes |
| 112 | 1OBP | ODORANT-BINDING PROTEIN FROM BOVINE NASAL MUCOSA | 2 | yes | yes |
| 113 | 1OM3 | FAB 2G12 unliganded | 2.2 | yes | yes |
| 114 | 1OP3 | Crystal Structure of Fab 2G12 bound to Man1->2Man | 1.75 | yes | yes |
| 115 | 1OP5 | Crystal Structure of Fab 2G12 bound to Man9GlcNAc2 | 3 | yes | yes |
| 116 | 1OQF | Crystal structure of the 2-methylisocitrate lyase | 1.93 | yes | yes |
| 117 | 1OSY | Crystal structure of FIP-Fve fungal immunomodulatory protein | 1.7 | yes | yes |
| 118 | 1OW6 | Paxillin LD4 motif bound to the Focal Adhesion Targeting (FAT) domain of the Focal Adhesion Kinase | 2.35 | yes | yes |
| 119 | 1OY0 | The crystal Structure of the First Enzyme of Pantothenate Biosynthetic Pathway, Ketopantoate Hydroxymethyltransferase from Mycobacterium Tuberculosis Shows a Decameric Assembly and Terminal Helix-Swapping | 2.8 | yes | yes |
| 120 | 1P3Q | Mechanism of Ubiquitin Recognition by the CUE Domain of VPS9 | 1.7 | yes | yes |
| 121 | 1P93 | CRYSTAL STRUCTURE OF THE AGONIST FORM OF GLUCOCORTICOID RECEPTOR | 2.7 | yes | yes |
| 122 | 1PUC | P13SUC1 IN A STRAND-EXCHANGED DIMER | 1.95 | yes | yes |
| 124 | 1PYM | PHOSPHOENOLPYRUVATE MUTASE FROM MOLLUSK IN WITH BOUND MG2-OXALATE | 1.8 | yes | yes |
| 126 | 1Q8M | Crystal structure of the human myeloid cell activating receptor TREM-1 | 2.6 | yes | yes |
| 127 | 1QB3 | CRYSTAL STRUCTURE OF THE CELL CYCLE REGULATORY PROTEIN CKS1 | 3 | yes | yes |
| 128 | 1QOM | MURINE INDUCIBLE NITRIC OXIDE SYNTHASE OXYGENASE DIMER (DELTA 65) WITH SWAPPED N-TERMINAL HOOK | 2.7 | yes | yes |
| 129 | 1QQ2 | CRYSTAL STRUCTURE OF A MAMMALIAN 2-CYS PEROXIREDOXIN, HBP23 | 2.6 | yes | yes |
| 130 | 1QWI | Crystal Structure of E. coli OsmC | 1.8 | yes | yes |
| 131 | 1QX5 | Crystal structure of apoCalmodulin | 2.54 | yes | yes |
| 132 | 1QX7 | Crystal structure of apoCaM bound to the gating domain of small conductance Ca2+-activated potassium channel | 3.09 | yes | yes |
| 133 | 1R4C | N-Truncated Human Cystatin C; Dimeric Form With 3D Domain Swapping | 2.18 | yes | yes |
| 134 | 1R5C | X-ray structure of the complex of Bovine seminal ribonuclease swapping dimer with d(CpA) | 2.1 | yes | yes |
| 135 | 1R5D | X-ray structure of bovine seminal ribonuclease swapping dimer from a new crystal form | 2.5 | yes | yes |
| 136 | 1R7H | NrdH-redoxin of Corynebacterium ammoniagenes forms a domain-swapped dimer | 2.69 | yes | yes |
| 137 | 1R8J | Crystal Structure of Circadian Clock Protein KaiA from Synechococcus elongatus | 2.03 | yes | yes |
| 138 | 1R9X | Bacterial cytosine deaminase D314G mutant. | 1.58 | yes | yes |
| 139 | 1R9Y | Bacterial cytosine deaminase D314A mutant. | 1.57 | yes | yes |
| 140 | 1R9Z | Bacterial cytosine deaminase D314S mutant. | 1.32 | yes | yes |
| 141 | 1S8O | Human soluble Epoxide Hydrolase | 2.6 | yes | yes |
| 142 | 1SB2 | High resolution Structure determination of rhodocetin | 1.9 | yes | yes |
| 143 | 1SCE | CRYSTAL STRUCTURE OF THE CELL CYCLE REGULATORY PROTEIN SUC1 REVEALS A NOVEL BETA-HINGE CONFORMATIONAL SWITCH | 2.2 | yes | yes |
| 144 | 1SGK | NUCLEOTIDE-FREE DIPHTHERIA TOXIN | 2.3 | yes | yes |
| 145 | 1SJV | Three-Dimensional Structure of a Llama VHH Domain Swapping | 1.94 | yes | yes |
| 146 | 1SND | STAPHYLOCOCCAL NUCLEASE DIMER CONTAINING A DELETION OF RESIDUES 114-119 COMPLEXED WITH CALCIUM CHLORIDE AND THE COMPETITIVE INHIBITOR DEOXYTHYMIDINE-3', 5'-DIPHOSPHATE | 1.84 | yes | yes |
| 147 | 1SR9 | Crystal Structure of LeuA from Mycobacterium tuberculosis | 2 | yes | yes |
| 148 | 1SVA | SIMIAN VIRUS 40 | 3.1 | yes | yes |
| 149 | 1T92 | Crystal structure of N-terminal truncated pseudopilin PulG | 1.6 | yes | yes |
| 150 | 1T98 | Crystal Structure of MukF(1-287) | 2.9 | yes | yes |
| 151 | 1TIJ | 3D Domain-swapped human cystatin C with amyloid-like intermolecular beta-sheets | 3.03 | yes | yes |
| 152 | 1TQ9 | Non-covalent swapped dimer of Bovine Seminal Ribonuclease in complex with 2'-DEOXYCYTIDINE-2'-DEOXYADENOSINE-3', 5'-MONOPHOSPHATE | 2 | yes | yes |
| 153 | 1TUW | Structural and Functional Analysis of Tetracenomycin F2 Cyclase from Streptomyces glaucescens: A Type-II Polyketide Cyclase | 1.9 | yes | yes |
| 154 | 1U0R | Crystal structure of Mycobacterium tuberculosis NAD kinase | 2.8 | yes | yes |
| 155 | 1U0T | Crystal structure of Mycobacterium tuberculosis NAD kinase | 2.3 | yes | yes |
| 156 | 1U4N | Crystal Structure Analysis of the M211S/R215L EST2 mutant | 2.1 | yes | yes |
| 157 | 1U7H | Structure and a Proposed Mechanism for Ornithine Cyclodeaminase from Pseudomonas putida | 1.8 | yes | yes |
| 158 | 1UKM | Crystal structure of EMS16, an Antagonist of collagen receptor integrin alpha2beta1 (GPIa/IIa) | 1.9 | yes | yes |
| 159 | 1VJ5 | Human soluble Epoxide Hydrolase- N-cyclohexyl-N'-(4-iodophenyl)urea complex | 2.35 | yes | yes |
| 160 | 1W58 | FTSZ GMPCPP SOAK I213 (M. JANNASCHII) | 2.5 | yes | yes |
| 161 | 1W5F | FTSZ T7 MUTATED, DOMAIN SWAPPED (T. MARITIMA) | 2 | yes | yes |
| 162 | 1WKQ | Crystal Structure of Bacillus subtilis Guanine Deaminase. The first domain-swapped structure in the cytidine deaminase superfamily | 1.17 | yes | yes |
| 163 | 1WWA | NGF BINDING DOMAIN OF HUMAN TRKA RECEPTOR | 2.5 | yes | yes |
| 164 | 1WWB | LIGAND BINDING DOMAIN OF HUMAN TRKB RECEPTOR | 2.1 | yes | yes |
| 165 | 1WWC | NT3 BINDING DOMAIN OF HUMAN TRKC RECEPTOR | 1.9 | yes | yes |
| 166 | 1X0G | Crystal Structure of IscA with the [2Fe-2S] cluster | 2.5 | yes | yes |
| 167 | 1X2W | Crystal Structure of Apo-Habu IX-bp at pH 4.6 | 2.29 | yes | yes |
| 168 | 1X7D | Crystal Structure Analysis of Ornithine Cyclodeaminase Complexed with NAD and ornithine to 1.6 Angstroms | 1.6 | yes | yes |
| 169 | 1XCB | X-ray Structure of a Rex-Family Repressor/NADH Complex from Thermus Aquaticus | 2.9 | yes | yes |
| 170 | 1XIU | Crystal structure of the agonist-bound ligand-binding domain of Biomphalaria glabrata RXR | 2.5 | yes | yes |
| 171 | 1XML | Structure of human Dcps | 2 | yes | yes |
| 172 | 1XMM | Structure of human Dcps bound to m7GDP | 2.5 | yes | yes |
| 173 | 1XUU | Crystal structure of sialic acid synthase (NeuB) in complex with Mn2+ and Malate from Neisseria meningitidis | 1.9 | yes | yes |
| 174 | 1XUZ | Crystal structure analysis of sialic acid synthase (NeuB)from Neisseria meningitidis, bound to Mn2+, Phosphoenolpyruvate and N-acetyl mannosaminitol | 2.2 | yes | yes |
| 175 | 1Y50 | X-ray crystal structure of Bacillus stearothermophilus Histidine phosphocarrier protein (Hpr) F29W mutant domain_swapped dimer | 2 | yes | yes |
| 177 | 1Y8N | Crystal structure of the PDK3-L2 complex | 2.6 | yes | yes |
| 178 | 1Y8O | Crystal structure of the PDK3-L2 complex | 2.48 | yes | yes |
| 179 | 1Y8P | Crystal structure of the PDK3-L2 complex | 2.63 | yes | yes |
| 180 | 1Y92 | Crystal structure of the P19A/N67D Variant Of Bovine seminal Ribonuclease | 2.2 | yes | yes |
| 181 | 1Y94 | Crystal structure of the G16S/N17T/P19A/S20A/N67D Variant Of Bovine seminal Ribonuclease | 2.2 | yes | yes |
| 183 | 1YGT | Dynein Light Chain TcTex-1 | 1.7 | yes | yes |
| 185 | 1YVS | Trimeric domain swapped barnase | 2.2 | yes | yes |
| 186 | 1ZD2 | Human soluble epoxide hydrolase 4-(3-cyclohexyluriedo)-ethanoic acid complex | 3 | yes | yes |
| 187 | 1ZD3 | Human soluble epoxide hydrolase 4-(3-cyclohexyluriedo)-butyric acid complex | 2.3 | yes | yes |
| 188 | 1ZD4 | Human soluble epoxide hydrolase 4-(3-cyclohexyluriedo)-hexanoic acid complex | 2.7 | yes | yes |
| 189 | 1ZD5 | Human soluble epoxide hydrolase 4-(3-cyclohexyluriedo)-heptanoic acid complex | 2.6 | yes | yes |
| 190 | 1ZLS | FAB 2G12 + Man4 | 2 | yes | yes |
| 191 | 1ZLU | FAB 2G12 + Man5 | 2.75 | yes | yes |
| 192 | 1ZLV | Fab 2G12 + Man7 | 2.33 | yes | yes |
| 193 | 1ZLW | Fab 2G12 + Man8 | 2.85 | yes | yes |
| 194 | 1ZV1 | Crystal structure of the dimerization domain of doublesex protein from D. melanogaster | 1.6 | yes | yes |
| 195 | 1ZVN | Crystal structure of chick MN-cadherin EC1 | 2.16 | yes | yes |
| 196 | 1ZXK | Crystal Structure of Cadherin8 EC1 domain | 2 | yes | yes |
| 197 | 2A07 | Crystal Structure of Foxp2 bound Specifically to DNA. | 1.9 | yes | yes |
| 198 | 2A4E | Crystal structure of mouse cadherin-11 EC1-2 | 3.2 | yes | yes |
| 199 | 2A5H | 2.1 Angstrom X-ray crystal structure of lysine-2, 3-aminomutase from Clostridium subterminale SB4 with Michaelis analog (L-alpha-lysine external aldimine form of pyridoxal-5'-phosphate) | 2.1 | yes | yes |
| 200 | 2A62 | Crystal structure of mouse cadherin-8 EC1-3 | 4.5 | yes | yes |
| 201 | 2A9U | Structure of the N-terminal domain of Human Ubiquitin carboxyl-terminal hydrolase 8 (USP8) | 2.1 | yes | yes |
| 202 | 2AHR | Crystal Structures of 1-Pyrroline-5-Carboxylate Reductase from Human Pathogen Streptococcus pyogenes | 2.15 | yes | yes |
| 203 | 2AK7 | structure of a dimeric P-Ser-Crh | 2 | yes | yes |
| 204 | 2AMF | Crystal structure of 1-Pyrroline-5-Carboxylate Reductase from Human Pathogen Streptococcus Pyogenes | 2.2 | yes | yes |
| 205 | 2AOA | Crystal structures of a high-affinity macrocyclic peptide mimetic in complex with the Grb2 SH2 domain | 1.99 | yes | yes |
| 206 | 2AOB | Crystal structures of a high-affinity macrocyclic peptide mimetic in complex with the Grb2 SH2 domain | 1.8 | yes | yes |
| 207 | 2B48 | Bcl-XL 3D Domain Swapped Dimer | 3.45 | yes | yes |
| 208 | 2BB2 | X-RAY ANALYSIS OF BETA B2-CRYSTALLIN AND EVOLUTION OF OLIGOMERIC LENS PROTEINS | 2.1 | yes | yes |
| 209 | 2BH8 | COMBINATORIAL PROTEIN 1B11 | 1.9 | yes | yes |
| 210 | 2BI4 | LACTALDEHYDE:1, 2-PROPANEDIOL OXIDOREDUCTASE OF ESCHERICHIA COLI | 2.85 | yes | yes |
| 211 | 2BL4 | LACTALDEHYDE:1, 2-PROPANEDIOL OXIDOREDUCTASE OF ESCHERICHIA COLI | 2.85 | yes | yes |
| 212 | 2C3B | THE CRYSTAL STRUCTURE OF ASPERGILLUS FUMIGATUS CYCLOPHILIN REVEALS 3D DOMAIN SWAPPING OF A CENTRAL ELEMENT | 1.85 | yes | yes |
| 213 | 2C5J | N-TERMINAL DOMAIN OF TLG1, DOMAIN-SWAPPED DIMER | 2.1 | yes | yes |
| 214 | 2CN4 | THE CRYSTAL STRUCTURE OF THE SECRETED DIMERIC FORM OF THE HEMOPHORE HASA REVEALS A DOMAIN SWAPPING WITH AN EXCHANGED HEME LIGAND | 2.3 | yes | yes |
| 215 | 2CO3 | SALMONELLA ENTERICA SAFA PILIN, HEAD-TO-TAIL SWAPPED DIMER OF NTD1 MUTANT | 1.78 | yes | yes |
| 216 | 2DI3 | Crystal structure of the transcriptional factor CGL2915 from Corynebacterium glutamicum | 2.05 | yes | yes |
| 217 | 2DSB | Crystal structure of human ADP-ribose pyrophosphatase NUDT5 | 2.5 | yes | yes |
| 218 | 2DSC | Crystal structure of human ADP-ribose pyrophosphatase NUDT5 in complex with magnesium and ADP-ribose | 2 | yes | yes |
| 219 | 2DSD | Crystal structure of human ADP-ribose pyrophosphatase NUDT5 in complex with magnesium and AMP | 2.6 | yes | yes |
| 220 | 2DWK | Crystal structure of the RUN domain of mouse Rap2 interacting protein x | 2 | yes | yes |
| 221 | 2DXL | Glycerophosphodiesterase from Enterobacter aerogenes | 3 | yes | yes |
| 222 | 2ES0 | Structure of the regulator of G-protein signaling domain of RGS6 | 2.1 | yes | yes |
| 224 | 2FPN | The crystal structure of the ywmB protein from Bacillus subtilis | 2.49 | yes | yes |
| 225 | 2FQM | Crystal structure of the oligomerization domain of the phosphoprotein of vesicular stomatitis virus | 2.3 | yes | yes |
| 226 | 2FZN | Structure of the E. coli PutA proline dehydrogenase domain reduced by dithionite and complexed with proline | 2 | yes | yes |
| 227 | 2GH8 | X-ray structure of a native calicivirus | 3.2 | yes | yes |
| 228 | 2GSY | The 2.6A structure of Infectious Bursal Virus Derived T=1 Particles | 2.6 | yes | yes |
| 229 | 2GTY | Crystal structure of unliganded griffithsin | 1.3 | yes | yes |
| 230 | 2GUC | Crystal structure of a complex of griffithsin with mannose at 1.78 A resolution. | 1.79 | yes | yes |
| 231 | 2GUD | Crystal structure of a complex of griffithsin with mannose at 0.94 A resolution | 0.94 | yes | yes |
| 232 | 2GUE | Crystal structure of a complex of griffithsin with N-acetylglucosamine | 2.02 | yes | yes |
| 233 | 2GUX | Selenomethionine derivative of griffithsin | 2 | yes | yes |
| 234 | 2GZA | Crystal structure of the VirB11 ATPase from the Brucella Suis type IV secretion system in complex with sulphate | 2.6 | yes | yes |
| 235 | 2H46 | Native domain-swapped dimer crystal structure of the Grb2 SH2 domain | 1.9 | yes | yes |
| 236 | 2H5K | Crystal Structure of Complex Between the Domain-Swapped Dimeric Grb2 SH2 Domain and Shc-Derived Ligand : Ac-NH-pTyr-Val-Asn-NH2 | 3.25 | yes | yes |
| 237 | 2HAX | Crystal structure of Bacillus caldolyticus cold shock protein in complex with hexathymidine | 1.29 | yes | yes |
| 238 | 2HJ1 | Crystal structure of a 3D domain-swapped dimer of protein HI0395 from Haemophilus influenzae | 2.1 | yes | yes |
| 239 | 2HKN | Crystal structure of the CAP-Gly domain of human Dynactin-1 (p150-Glued) | 1.87 | yes | yes |
| 240 | 2HN1 | Crystal structure of a CorA soluble domain from A. fulgidus in complex with Co2+ | 2.9 | yes | yes |
| 241 | 2HSN | Structural basis of yeast aminoacyl-tRNA synthetase complex formation revealed by crystal structures of two binary sub-complexes | 2.2 | yes | yes |
| 242 | 2HYQ | Crystal structure of a complex of griffithsin with 6alpha-mannobiose | 2 | yes | yes |
| 243 | 2HYR | Crystal structure of a complex of griffithsin with maltose | 1.51 | yes | yes |
| 244 | 2HZK | Crystal structures of a sodium-alpha-keto acid binding subunit from a TRAP transporter in its open form | 1.7 | yes | yes |
| 245 | 2HZL | Crystal structures of a sodium-alpha-keto acid binding subunit from a TRAP transporter in its closed forms | 1.4 | yes | yes |
| 246 | 2IEY | Crystal Structure of mouse Rab27b bound to GDP in hexagonal space group | 3.18 | yes | No |
| 247 | 2IF0 | Crystal Structure of mouse Rab27b bound to GDP in monoclinic space group | 2.8 | yes | yes |
| 248 | 2IV0 | THERMAL STABILITY OF ISOCITRATE DEHYDROGENASE FROM ARCHAEOGLOBUS FULGIDUS STUDIED BY CRYSTAL STRUCTURE ANALYSIS AND ENGINEERING OF CHIMERS | 2.5 | yes | yes |
| 249 | 2J6G | FAEG FROM F4AC ETEC STRAIN 5_95, PRODUCED IN TOBACCO PLANT CHLOROPLAST | 1.55 | yes | yes |
| 250 | 2J6R | FAEG FROM F4AC ETEC STRAIN GIS26, PRODUCED IN TOBACCO PLANT CHLOROPLAST | 1.9 | yes | yes |
| 252 | 2NSN | Crystal structure of Caspace Activation and Recruitment Domain (CARD) of NOD1 | 2 | yes | yes |
| 253 | 2NU5 | Crystal structure of a complex of griffithsin cocrystallized with N-acetylglucosamine | 1.56 | yes | yes |
| 254 | 2NUO | Crystal structure of a complex of griffithsin with glucose | 1.5 | yes | yes |
| 255 | 2NZ7 | Crystal Structure Analysis of Caspase-recruitment Domain (CARD) of Nod1 | 1.9 | yes | yes |
| 256 | 2OCT | Stefin B (Cystatin B) tetramer | 1.4 | yes | yes |
| 257 | 2ONT | A swapped dimer of the HIV-1 capsid C-terminal domain | 2.4 | yes | yes |
| 258 | 2OQR | The structure of the response regulator RegX3 from Mycobacterium tuberculosis | 2.03 | yes | yes |
| 260 | 2OYA | Crystal structure analysis of the dimeric form of the SRCR domain of mouse MARCO | 1.77 | yes | yes |
| 261 | 2P1J | Crystal structure of a polC-type DNA polymerase III exonuclease domain from Thermotoga maritima | 2.5 | yes | yes |
| 262 | 2P1L | Structure of the Bcl-XL:Beclin 1 complex | 2.5 | yes | yes |
| 263 | 2P67 | Crystal structure of LAO/AO transport system kinase | 1.8 | yes | yes |
| 264 | 2PA7 | Structure of Wild-Type dTDP-4-keto-6-deoxy-D-glucose-3, 4-ketoisomerase from Aneurinibacillus thermoaerophilus in complex with TDP | 1.5 | yes | yes |
| 265 | 2PAE | Structure of a H49N mutant dTDP-4-keto-6-deoxy-D-glucose-3, 4-ketoisomerase from Aneurinibacillus thermoaerophilus in complex with TDP | 2.5 | yes | yes |
| 266 | 2PAH | TETRAMERIC HUMAN PHENYLALANINE HYDROXYLASE | 3.1 | yes | yes |
| 267 | 2PAK | Structure of a H51N mutant dTDP-4-keto-6-deoxy-D-glucose-3, 4-ketoisomerase from Aneurinibacillus thermoaerophilus complexed with TDP | 2.4 | yes | yes |
| 268 | 2PAM | Structure of a H49N, H51N double mutant dTDP-4-keto-6-deoxy-D-glucose-3, 4-ketoisomerase from Aneurinibacillus thermoaerophilus complexed with TDP | 2.5 | yes | yes |
| 269 | 2PJW | The Vps27/Hse1 complex is a GAT domain-based scaffold for ubiquitin-dependent sorting | 3.01 | yes | yes |
| 270 | 2PQM | Crystal structure of Cysteine Synthase (OASS) from Entamoeba histolytica at 1.86 A resolution | 1.86 | yes | yes |
| 271 | 2QDN | Crystal Structure of mouse GITRL | 2.09 | yes | yes |
| 272 | 2QYP | Orthorhombic Crystal Structure of Human Saposin C Dimer in Open Conformation | 2.45 | yes | yes |
| 273 | 2RCZ | Structure of the second PDZ domain of ZO-1 | 1.7 | yes | yes |
| 274 | 2SPC | CRYSTAL STRUCTURE OF THE REPETITIVE SEGMENTS OF SPECTRIN | 1.8 | yes | yes |
| 275 | 2VAJ | CRYSTAL STRUCTURE OF NCAM2 IG1 (I4122 CELL UNIT) | 2.7 | yes | yes |
| 276 | 2VTY | VACCINIA VIRUS ANTI-APOPTOTIC F1L IS A NOVEL BCL-2-LIKE DOMAIN SWAPPED DIMER | 2.1 | yes | yes |
| 277 | 2W1T | CRYSTAL STRUCTURE OF B. SUBTILIS SPOVT | 2.6 | yes | yes |
| 278 | 2Z4H | Crystal structure of the Cpx pathway activator NlpE from Escherichia coli | 2.8 | yes | yes |
| 279 | 2Z4I | Crystal structure of the Cpx pathway activator NlpE from Escherichia coli | 2.6 | yes | yes |
| 280 | 2Z9A | Crystal Structure of Human Saposin C Dimer in Open Conformation | 2.5 | yes | yes |
| 281 | 2ZEJ | Structure of the ROC domain from the Parkinson's disease-associated leucine-rich repeat kinase 2 reveals a dimeric GTPase | 2 | yes | yes |
| 282 | 2ZNH | Crystal Structure of a Domain-Swapped Serpin Dimer | 2.8 | yes | yes |
| 283 | 3B9I | Crystal Structure of mouse GITRL at 2.5 A. | 2.49 | yes | yes |
| 284 | 3BCO | Crystal Structure of The Swapped FOrm of P19A/L28Q/N67D BS-RNase | 2.25 | yes | yes |
| 285 | 3BCP | Crystal Structure of The Swapped non covalent form of P19A/L28Q/N67D BS-RNase | 2.57 | yes | No |
| 286 | 3BM5 | Crystal structure of O-acetyl-serine sulfhydrylase from Entamoeba histolytica in complex with cysteine | 2.4 | yes | yes |
| 287 | 3CYY | The crystal structure of ZO-1 PDZ2 in complex with the Cx43 peptide | 2.4 | yes | yes |
| 288 | 3D6T | Structure of the ROC domain from the Parkinson's disease-associated leucine-rich repeat kinase 2 reveals a dimeric GTPase | 2.43 | yes | yes |
| 289 | 3EOT | Crystal structure of LAC031, an engineered anti-VLA1 Fab | 1.9 | yes | yes |
| 290 | 3EZM | CYANOVIRIN-N | 1.5 | yes | yes |
| 291 | 3FIG | Crystal Structure of Leucine-bound LeuA from Mycobacterium tuberculosis | 2.3 | yes | yes |
| 292 | 5CRO | REFINED STRUCTURE OF CRO REPRESSOR PROTEIN FROM BACTERIOPHAGE LAMBDA | 2.3 | yes | yes |
| 293 | 7CAT | THE NADPH BINDING SITE ON BEEF LIVER CATALASE | 2.5 | yes | yes |
